# Investigating the association between birth weight and complementary air pollution metrics: a cohort study

**DOI:** 10.1186/1476-069X-12-18

**Published:** 2013-02-17

**Authors:** Olivier Laurent, Jun Wu, Lianfa Li, Judith Chung, Scott Bartell

**Affiliations:** 1 Program in Public Health, University of California, Irvine, CA, USA; 2 State Key Lab of Resources and Environmental Information Systems, Institute of Geographical Sciences and Natural Resources Research, Chinese Academy of Sciences, Beijing, China; 3 Division of Maternal Fetal Medicine, University of California, Irvine, CA, USA; 4 Department of Epidemiology, University of California, Irvine, CA, USA; 5 Department of Statistics, University of California, Irvine, CA, USA

**Keywords:** Birth weight, Air pollution, Exposure assessment, Traffic, Ozone

## Abstract

**Background:**

Exposure to air pollution is frequently associated with reductions in birth weight but results of available studies vary widely, possibly in part because of differences in air pollution metrics. Further insight is needed to identify the air pollution metrics most strongly and consistently associated with birth weight.

**Methods:**

We used a hospital-based obstetric database of more than 70,000 births to study the relationships between air pollution and the risk of low birth weight (LBW, <2,500 g), as well as birth weight as a continuous variable, in term-born infants. Complementary metrics capturing different aspects of air pollution were used (measurements from ambient monitoring stations, predictions from land use regression models and from a Gaussian dispersion model, traffic density, and proximity to roads). Associations between air pollution metrics and birth outcomes were investigated using generalized additive models, adjusting for maternal age, parity, race/ethnicity, insurance status, poverty, gestational age and sex of the infants.

**Results:**

Increased risks of LBW were associated with ambient O_3_ concentrations as measured by monitoring stations, as well as traffic density and proximity to major roadways. LBW was not significantly associated with other air pollution metrics, except that a decreased risk was associated with ambient NO_2_ concentrations as measured by monitoring stations. When birth weight was analyzed as a continuous variable, small increases in mean birth weight were associated with most air pollution metrics (<40 g per inter-quartile range in air pollution metrics). No such increase was observed for traffic density or proximity to major roadways, and a significant decrease in mean birth weight was associated with ambient O_3_ concentrations.

**Conclusions:**

We found contrasting results according to the different air pollution metrics examined. Unmeasured confounders and/or measurement errors might have produced spurious positive associations between birth weight and some air pollution metrics. Despite this, ambient O_3_ was associated with a decrement in mean birth weight and significant increases in the risk of LBW were associated with traffic density, proximity to roads and ambient O_3_. This suggests that in our study population, these air pollution metrics are more likely related to increased risks of LBW than the other metrics we studied. Further studies are necessary to assess the consistency of such patterns across populations.

## Background

Limitation of intrauterine growth has important consequences for infant, child and adult health. Associations have been reported between intrauterine growth restriction and the incidence of several chronic conditions in later life such as type 2 diabetes mellitus [1] or cardiovascular diseases [2]. It has been hypothesized that variations in birth weight are among the most visible manifestations of a broader set of biological changes setting grounds for the development of non-communicable diseases in later life and that such biological changes may be caused, noticeably, by environmental factors [3]. Air pollution stands among the environmental factors suspected to hamper intrauterine growth. Hypothesized mechanisms include impaired placental oxygen and nutrient transport to the fetus, as possible consequences of well-established biological changes induced by exposure to air pollution (such as systemic oxidative stress, inflammation, perturbed endothelial function, blood coagulation and viscosity or hemodynamic responses) [4], or less explored pathways such as endocrine disruption, genetic or epigenetic changes [3,5].

In order to confront these biological hypotheses with real-world observations, a rapidly increasing number of epidemiological studies has investigated the relations between exposure to air pollution and fetal growth measured in utero [6,7] or resulting birth weight [8,9]. While most available studies suggest that air pollution may produce adverse impacts on these outcomes, results vary widely, both quantitatively and qualitatively, according to study settings and methodologies [10,11]. Some authors stress the fact that heterogeneity in air pollution exposure metrics (along with other differences, such as adjustment for different sets of potential confounders) may significantly contribute to the observed differences in study results [12]. Efforts are underway at an international scale to provide pooled results from existing studies using more harmonized analytical strategies [11]. In parallel, additional insight into the impact of exposure assessment methods on the relation between air pollution and birth outcomes is warranted [13].

In the present work, we studied the relationships between birth weight and air pollution, characterized using complementary metrics: measurements from ambient monitoring stations, predictions from dispersion and land use regression (LUR) models, traffic density, and distance to roadways.

## Methods

### Study setting and population

We obtained birth data for period 1997–2006 from the Memorial Care System, a network of four hospitals maintaining a perinatal database for research purposes. These hospitals (Anaheim, Long Beach, Orange Coast and Saddleback Memorial Medical Centers) are located in Los Angeles and Orange counties, Southern California, USA. Together, both counties cover an area of 160*161 km^2^ and were home to approximately 12 million people in year 2000 [14]. Only infants born to mothers residing in Los Angeles and Orange counties at the time of delivery were included in the study.

A total of 105,092 neonatal records were extracted from the database. Geocoding of the residential address at delivery was conducted using the TeleAtlas Geocoding Service [15] with a 93% success rate. Multiple gestations were excluded (5%) as well as subjects with unsuccessfully geocoded addresses, missing addresses or missing important covariate information used in previous studies (12%) [13,16]. We excluded subjects born before 37 or after 44 weeks of gestation (8%), in order to study the influence of air pollution on term birth weight only. Preterm birth might have a different etiology and was addressed in our previous publications using the same database [13,16].

### Individual variables

Individual variables recorded in the hospital database included birth weight and gender of the infant, maternal age, race/ethnicity, parity (primiparous or not), whether the mother received prenatal care during pregnancy (yes/no) and indicator variables for gestational weight gain during pregnancy (low <15 lbs (6.8 kg) and high >30 lbs (13.6 kg)). Maternal conditions during pregnancy associated with birth weight (diabetes, hypertension, heart diseases, preeclampsia) were also recorded. The length of gestation was estimated using the date of last menstrual period and ultrasound dating: the date of last menstrual period was used, unless it differed from ultrasound dating estimates by at least 1 week in the first trimester, 2 weeks in the second trimester or 3 weeks in the third trimester. In such cases, ultrasound-based estimates were used.

### Neighborhood socioeconomic and demographic variables

We used maternal addresses to link them to the Census Block Groups in which they were located. For each of these Block Groups, we calculated the following indicators using variables from the 2000 Census: population density (number of inhabitants/km^2^), median income, and poverty (defined as the % of population living below the federal poverty line) [14].

### Air pollution metrics

Epidemiological studies of air pollution and birth outcomes ideally use individual estimates of maternal exposure to air pollution during pregnancy [17]. However, such estimates are still difficult to produce for large scale or retrospective studies in which no time-activity information or estimates of pollutant concentrations in micro-environments are available. We therefore used five categories of proxy air pollution metrics in this study, namely: 1) measurements from ambient monitoring stations, 2) predictions from a dispersion model, 3) predictions from land use regression (LUR) models, 4) traffic density, and 5) distance to roadways. Monitor-based measurements provide the greatest temporal variability and reflect mostly regional emission sources (and to a lesser extent, local sources especially when monitors are surrounded by a high density of these, e.g. in Los Angeles County). The dispersion model predictions reflect local traffic emissions at a high spatial resolution but with a limited temporal variability. LUR predictions capture local traffic emissions but also local land use characteristics as well as regional traffic emissions. Traffic density and distance to roadways are crude proxies for primary emissions of traffic-related pollutants, but are easy to obtain and have been used in many epidemiological studies. These air pollution metrics have already been used in a previous epidemiological study of preeclampsia and preterm birth conducted in the same setting [13], although the present study further presents results according to distances to roadways.

#### Ambient monitoring data

Air pollution measurements were obtained from the California Air Resources Board [18] for nitrogen dioxide (NO_2_), nitric oxide (NO), nitrogen oxides (NO_x_), carbon monoxide (CO), ozone (O_3_), particulate matter of less than 10 μm and less than 2.5 μm in aerodynamic diameter (PM_10_ and PM_2.5_, respectively). Ambient pollutant concentrations were attributed to each mother according to the “nearest station approach”. We assigned pollutant concentrations measured at the operational monitoring station closest to a mother’s home. The number of active monitoring stations varied by pollutant and by year. During 1997–2006 in the study area, there were 17–21 stations with valid measurement data for NO, NO_2_, NO_x_ and O_3_, 14–19 stations for CO and 10–11 stations for PM_10_. An exception was PM_2.5_ data for which a sufficient number of monitors (9–13) were operational only from year 2000. Hourly measurements of NO_2_, NO_x_, NO, CO, and O_3_ were converted to daily means using a criterion of 75% daily data completeness. Only data for the 10 AM – 6 PM time windows were used to calculate daily means for O_3_, while these were based on 24 hours for other pollutants. Monthly averages for gaseous pollutants were then calculated for stations with more than 22 days of valid data in a month. For PM, we only included filter based daily measurements that were collected every 3rd or 6th day. Monthly averages for PM_10_ and PM_2.5_ were calculated if three or more daily measurements per month were available. Final pollutant concentration estimates were calculated for each mother by weighting monthly average concentrations by the number of days in each month for specific pregnancy periods (entire pregnancy period, 1st, 2nd or 3rd trimester). Further details on the procedure can be found in another article [13].

#### Land use regression models

LUR models were developed for NO_2_ and NO_x_ ambient concentrations, based on simultaneous two-week measurements during September 2006 and February 2007, using Ogawa passive diffusion samplers at 181 sites in Los Angeles [19]. The LUR models included the variables vehicle miles traveled on highways, major and other roads, total lengths of major and local road segments, land use data (industrial, commercial) and satellite-derived soil brightness within circular buffers of various radii centered on the sampling sites, distance between the sampling sites and nearest truck routes, and coordinates of the sampling sites. The final regression models explained 86% and 85% of the variance in measured NO_2_ and NO_x_ concentrations, respectively [19].

From predicted annual pollution surfaces, we derived temporally-adjusted LUR estimates based on the relative temporal profiles of yearly and monthly concentrations of pollutants measured at ambient monitoring stations. Temporally adjusted monthly LUR concentrations at each residence were calculated by multiplying the unadjusted annual LUR estimates by the yearly and monthly scaling factors at the nearest monitoring station, and then averaging over the pregnancy periods [13].

#### CALINE 4 dispersion model

We used a modified version of CALINE 4 Gaussian dispersion model [20] to predict ambient concentrations resulting from local traffic emissions of several pollutants (NO_x_, CO, PM_10_ and PM_2.5_) up to 3 km from maternal homes. Input data for the prediction process included roadway geometry and annual average daily traffic flow from California Department of Transportation (CALTRANS). The data cover all freeways and highways and most major arterial streets and were derived from a combination of tri-annual measurements and estimated values. The source traffic data obtained from CALTRANS were estimated for year 2005. We then scaled their values to the 1997–2006 trend in vehicle miles traveled in Los Angeles and Orange counties, in order to derive yearly traffic flow estimates across the study period. Other inputs to the CALINE4 model included emission factors from the California Air Resources Board’s EMFAC2007 vehicle emissions model [21] and hourly meteorological parameters (wind speed, wind direction, temperature stability class, and mixing heights). CALINE4 predictions in this study did not incorporate background levels of pollutants, thus solely represents the contribution from local traffic emissions. Further details about the modeling process can be found in an open-access publication [16]. CALINE4 predictions for PM_2.5_ and PM_10_ averaged over the entire pregnancy period were highly correlated (r> 0.99), we therefore report results only for PM_2.5_ CALINE4 predictions in this article.

#### Traffic density

Traffic densities were calculated within buffers covering different perpendicular distances from roads, using the kernel density plotting for line features of the Spatial Analyst extension in ArcInfoGIS9.1 (ESRI, Redlands, CA). The source of data for roadway geometry and traffic flow was CALTRANS annual average daily traffic counts for year 2005 (a scaling similar as above to the trend in vehicle miles traveled during the study period did not notably modify the results of the epidemiological analyses). Estimated traffic densities decrease from traffic-volume-dependent values at roadway edges, to zero at perpendicular distances from roads exceeding the defined buffer widths. The traffic density at each residential location was the sum of the traffic density from contributing roadway segments. We studied varying buffer sizes to allow for varying spreading distances for different pollutant or mixtures (50, 75, 100, 150, 200, 300 meters).

#### Distance to roadways

We obtained detailed roadway data from the 2003 TeleAtlas® street polylines database. We calculated the distance to the nearest freeway (defined by categories of the U.S. Census Feature Class Codes A10-A19), and to the nearest main road (A20-A39, standing for “primary roads without limited access, U.S., State and County highway”, and “secondary and connecting roads”).

### Statistical analyses

We used generalized additive models (GAMs) as implemented in the ‘mgcv’ package of the R environment (version 2.15.0) to study the relations between air pollution metrics and 1) term low birth weight (LBW) as a dichotomous variable (defined as birth weights inferior to 2,500 g) and 2) term birth weight as a continuous variable. Models were adjusted for potential confounders selected on the basis of previous knowledge and descriptive data analyses. In the main models, maternal age, length of gestation and poverty were adjusted for using smoothing splines, given their non-linear associations with the birth weight outcomes. Maternal race/ethnicity, insurance status, parity and infant’s gender were introduced as categorical variables.

Introducing air pollution metrics in the models using penalized smoothing splines with a limited upper number of degrees of freedom (less than 9, the effective number of degrees of freedom for the smooth being determined for each air pollution metric as part of model fitting via generalized cross validation) suggested no major departure from linearity in the relation between air pollution metrics and either LBW or birth weight (continuous variable). We therefore introduced air pollution metrics as linear terms in the models. We report related parameters estimates (i.e., either odds ratios for LBW or change in mean birth weight) for an inter-quartile range in air pollution metrics, with 95% confidence intervals and associated p-values.

Diagnostic plots for the GAMs modeling continuous birth weight as a dependent variable revealed that the distributions of residuals were closer to Student’s t, than to postulated normal, distributions. However, relaxing the normality assumption using the quasi family with constant variance did not lead to any noticeable differences in the results. We explored the use of multiple imputation techniques (20 simulations) using the ‘mi’ statistical package [15] to impute missing values for variables race/ethnicity (4%) and insurance status (4%). Since parameter estimates changed by less than 5% and the conclusions of the study remained unchanged, we present results based on complete-case analyses.

As part of sensitivity analyses, we examined the effects of adjustment for some variables that were not introduced in the main models (maternal heart diseases, diabetes, hypertension, preeclampsia, weight gain during pregnancy, population density). We also examined the effects of adjustment for month and year of conception and for either hospital or county. We explored the use of population density, as well as median income at the Census Block Group resolution, instead of poverty. Last, models introducing air pollutant concentrations averaged by pregnancy trimester one at a time were also used for the monitoring station measurements.

This study has been approved by the Institutional Review Board of the University of California, Irvine.

## Results

Table 1 describes the demographically and socioeconomically diverse study population. The birth weight indicators follow expected patterns according to maternal characteristics and infants’ gender. Table 2 shows descriptive statistics for the air pollution indicators corresponding to the whole pregnancy period [see Additional file 1 for graphical representations of their distributions], and correlations between them. Monitoring stations measurements for the different pollutants are overall highly positively correlated with each other, especially for gaseous pollutants (except for O_3_ which is strongly negatively correlated with the other pollutants) and the two fractions of particulate matter (PM_10_ and PM_2.5_). CALINE4 predictions are also highly correlated with each other. LUR predictions for NO_2_ and NO_x_ are relatively highly correlated with measurements from monitoring stations for the same pollutants (R=0.57 and 0.45, respectively) and highly correlated with each other (R=0.83). CALINE4 predictions are quite highly correlated with monitoring station measurements, and with LUR predictions (although less so for PM_2.5_). Traffic density is not correlated with monitoring station measurements and weakly so with LUR predictions. The correlations between traffic density and CALINE4 predictions increase as the buffer distance used for the calculation of traffic density increases (reaching 0.5-0.6 at 300 m). Distance to the nearest major road is weakly correlated with all other indicators, including with the distance to the nearest freeway. This last variable is also weakly correlated with all other indicators, except quite high negative correlations with CALINE4 predictions (between −0.48 and −0.55).

**Table 1:** Characteristics of the study population of term born infants and their mothers

**Subject characteristics**	**Number of subjects**	**Mean birth weight (g)**	**Standard deviation in birth weight (g)**	**% of infants with a low birth weight (<2,500 g)**
**Infant’s gender**				
**Female**	36,133	3,408.2	459.7	2.09
**Male**	38,283	3,527.5	473.5	1.35
**Race/ethnicity of mothers**				
**AfricanAm**	6,261	3,329.7	475.2	3.19
**Asian**	7,358	3,314.7	444.2	2.57
**Hispanic**	23,678	3,469.0	461.7	1.59
**Caucasian**	30,548	3,542.4	464.4	1.23
**Mixed**	322	3,473.2	458.2	0.93
**Other**	3,095	3,413.4	483.2	2.23
**Missing**	3,154	3,462.3	479.8	1.84
**Insurance status of mothers**				
**Private**	50,880	3,490.7	468.1	1.53
**Public**	20,525	3,418.5	475.9	2.2
**Missing**	3,011	3,460.2	450.3	1.3
**Chronic hypertension in mothers**				
**No**	74,138	3,469.8	470.3	1.7
**Yes**	278	3,395.9	551.7	3.96
**Heart diseases in mothers**				
**No**	74,333	3,469.7	470.6	1.7
**Yes**	83	3,309.7	511.1	6.02
**Preeclampsia in mothers**				
**No**	72,760	3,473.0	467.6	1.58
**Yes**	1656	3,319.4	570.2	7.37
**Diabetes in mothers**				
**No**	70,622	3,463.5	465.6	1.73
**Yes**	3794	3,582.5	544.6	1.34
**Maternal weight gain during pregnancy**				
**Low (<15 lbs)**	1286	3,376.1	491.1	3.34
**Medium**	57,727	3,445.9	463.2	1.79
**High (>30 lbs)**	15,397	3,566.1	483.5	1.24
**Missing**	6	2,978.8	686.4	0.08
**Parity**				
**First delivery**	60,382	3,458.3	471.4	1.81
**At least second delivery**	14,034	3,518.0	464.4	1.26

**Table 2:** Means, standard deviations and Pearson’s correlation coefficients for the air pollution metrics

					**Monitoring station measurements**							**Land Use Regression model predictions**	
		**N**	**Mean**	**Standard deviation**	**NO**_**2**_	**NO**	**NO**_**x**_	**CO**	**PM**_**10**_	**PM**_**2.5**_	**O**_**3**_	**NO**_**2**_	**NO**_**x**_
**Monitoring station measurements (a)**	**NO**_**2**_	74416	24.78	7.38	1.00								
	**NO**	74416	30.69	15.25	0.83	1.00							
	**NO**_**x**_	74416	55.42	21.74	0.92	0.98	1.00						
	**CO**	74416	0.73	0.36	0.83	0.85	0.88	1.00					
	**PM**_**10**_	74416	32.68	5.68	0.70	0.52	0.60	0.61	1.00				
	**PM**_**2.5**_	67141	17.47	3.48	0.77	0.65	0.71	0.77	0.87	1.00			
	**O**_**3**_	74416	35.66	7.41	−0.81	−0.80	−0.83	−0.74	−0.49	−0.61	1.00		
**Land Use Regression model predictions (a)**	**NO**_**2**_	74416	28.03	6.92	0.57	0.42	0.49	0.43	0.52	0. 52	−0.39	1.00	
	**NO**_**x**_	74416	59.93	20.07	0.37	0.46	0.45	0.35	0.37	0.38	−0.34	0.83	1.00
**CALINE4 predictions (a)**	**NO**_**x**_	72969	7.18	5.20	0.50	0.41	0.45	0.43	0.41	0.43	−0.37	0.63	0.49
	**CO**	72969	0.10	0.07	0.53	0.42	0.48	0.45	0.44	0.46	−0.38	0.64	0.49
	**PM**_**2.5**_	72969	4.25	3.11	0.25	0.21	0.23	0.21	0.19	0.20	−0.14	0.44	0.34
**Traffic density, within buffers of different distances around roads (b)**	**50 m**	74413	66.11	163.02	0.06	0.05	0.05	0.06	0.07	0.07	−0.04	0.10	0.09
	**75 m**	74413	56.56	132.89	0.06	0.04	0.05	0.05	0.06	0.06	−0.03	0.11	0.11
	**100 m**	74413	54.19	121.71	0.06	0.04	0.05	0.05	0.06	0.06	−0.03	0.13	0.13
	**150 m**	74413	58.33	119.27	0.07	0.05	0.06	0.06	0.07	0.07	−0.04	0.17	0.17
	**200 m**	74413	64.18	119.54	0.08	0.06	0.07	0.07	0.08	0.08	−0.05	0.21	0.21
	**250 m**	74413	69.29	118.22	0.08	0.06	0.07	0.07	0.08	0.08	−0.05	0.24	0.24
	**300 m**	74413	73.42	116.13	0.09	0.06	0.08	0.08	0.09	0.09	−0.05	0.27	0.27
**Distance to the nearest road (c)**	**Major roads**	74416	256.47	241.03	−0.15	−0.12	−0.14	−0.11	−0.12	−0.12	0.13	−0.15	−0.12
	**Freeways**	74416	1681.23	1244.81	−0.09	−0.07	−0.08	−0.07	−0.02	−0.04	0.05	−0.44	−0.32
		**CALINE4 predictions**			**Traffic density, within buffers of different distances around roads**							**Distance to the nearest road**	
		**NO**_**x**_	**CO**	**PM**_**2.5**_	**50 m**	**75 m**	**100 m**	**150 m**	**200 m**	**250 m**	**300 m**	**Major roads**	**Freeways**
**Monitoring station measurements (a)**	**NO**_**2**_												
	**NO**												
	**NO**_**x**_												
	**CO**												
	**PM**_**10**_												
	**PM**_**2.5**_												
	**O**_**3**_												
**Land Use Regression model predictions (a)**	**NO**_**2**_												
	**NO**_**x**_												
**CALINE4 predictions (a)**	**NO**_**x**_	1.00											
	**CO**	0.99	1.00										
	**PM**_**2.5**_	0.89	0.84	1.00									
**Traffic density, within buffers of different distances around roads (b)**	**50 m**	0.12	0.13	0.12	1.00								
	**75 m**	0.17	0.17	0.17	0.93	1.00							
	**100 m**	0.24	0.24	0.25	0.79	0.94	1.00						
	**150 m**	0.37	0.36	0.39	0.54	0.70	0.87	1.00					
	**200 m**	0.46	0.45	0.49	0.39	0.53	0.70	0.94	1.00				
	**250 m**	0.53	0.51	0.56	0.30	0.42	0.58	0.84	0.97	1.00			
	**300 m**	0.58	0.56	0.61	0.24	0.35	0.49	0.75	0.90	0.98	1.00		
**Distance to the nearest road (c)**	**Major roads**	−0.16	−0.17	−0.14	−0.26	−0.26	−0.24	−0.21	−0.19	−0.18	−0.17	1.00	
	**Freeways**	−0.51	−0.48	−0.55	−0.01	−0.03	−0.06	−0.12	−0.17	−0.21	−0.25	0.09	1.00

a) The units are parts per million (ppm) for CO, parts per billion (ppb) for NO, NO_2_, NOx, and O_3_, and μg.m^-3^ for PM_10_ and PM_2.5_. Concentrations are averages, across the pregnancy period, derived from daily 24 h- mean concentrations for NO_2_, NO, NO_x_, CO, PM_10_ and PM_2.5_ and from daily mean concentrations from 10 AM to 6 PM for O_3_.

b) The unit for traffic density is vehicle number per day/meter.

c) The unit for distance to road is meter.

When term LBW is analyzed as a binary outcome and monitoring station measurements are considered as air pollution metrics, a significant increase in risk is associated with O_3_ ambient concentrations. Conversely, decreases in risk (respectively, significant and close to significance) are associated with NO_2_ and NO_x_ concentrations (Table 3). No significant association is observed for the other pollutants measured by monitoring stations, for LUR or CALINE4 predictions, or for distance to freeways. In contrast, a decrease in the risk of LBW as distance to major roads increases is close to statistical significance (p=0.07). Beside, traffic density within buffers of 150 to 250 m from the roadways, is associated with an increased risk of LBW. For a 100 m distance, results are close to statistical significance (p=0.06). Similar associations are observed when women living outside the buffers (thus considered “non-exposed”) are excluded from analyses [see Additional file 2].

**Table 3:** Associations between birth weight and an inter-quartile range in air pollution metrics, for pregnancy-long exposures (a)

			**Low birth weight**				**Mean birth weight in grams**			
**Air pollution metrics**	**Inter-quartile range (IQR) in air pollution metrics**	**Number of subjects**	**Odds ratio per IQR increase in air pollution metrics**	**95% confidence interval**		**p value**	**Change for per IQR increase in air pollution metrics**	**95% confidence interval**		**p value**
**Monitoring station measurements, nearest station approach without distance restrictions (b)**										
**NO**_**2**_	11.87	68,303	0.85	0.76	0.94	< 0.01	36.24	30.50	41.98	< 0.01
**NO**_**x**_	27.70	68,303	0.93	0.86	1.00	0.06	25.31	20.84	29.78	< 0.01
**NO**	17.90	68,303	0.95	0.89	1.02	0.20	20.27	16.23	24.32	< 0.01
**CO**	0.48	68,303	0.96	0.88	1.04	0.28	22.79	18.23	27.35	< 0.01
**PM**_**10**_	6.76	68,303	0.94	0.87	1.01	0.09	20.74	16.68	24.79	< 0.01
**PM**_**2.5**_	5.10	61,623	0.93	0.84	1.02	0.11	26.83	21.56	32.11	< 0.01
**O**_**3**_	11.50	68,303	1.13	1.02	1.25	0.01	−31.36	−36.82	−25.89	< 0.01
**Land Use Regression model predictions (b)**										
**NO**_**2**_	9.34	68,303	0.94	0.86	1.02	0.13	16.59	12.01	21.16	< 0.01
**NO**_**x**_	25.24	68,303	0.98	0.91	1.06	0.66	9.42	5.25	13.59	< 0.01
**CALINE4 predictions (b)**										
**NO**_**x**_	5.65	67,043	0.97	0.90	1.04	0.35	14.10	10.29	17.91	< 0.01
**CO**	0.08	67,043	0.96	0.90	1.04	0.31	15.09	11.27	18.91	< 0.01
**PM**_**2.5**_	1.36	67,043	0.99	0.92	1.06	0.74	5.94	2.32	9.57	< 0.01
**Traffic density, within buffers of different distances around roads (b)**										
**50 m**	12.91	68,303	1.00	1.00	1.01	0.52	0.06	−0.21	0.32	0.68
**75 m**	35.91	68,303	1.01	0.99	1.02	0.24	−0.21	−1.12	0.69	0.64
**100 m**	53.91	68,303	1.02	1.00	1.05	0.06	−0.51	−2.00	0.97	0.50
**150 m**	74.30	68,303	1.05	1.01	1.08	0.01	−0.34	−2.43	1.75	0.75
**200 m**	84.33	68,303	1.05	1.01	1.09	0.01	0.06	−2.29	2.42	0.96
**250 m**	81.34	68,303	1.04	1.00	1.08	0.05	0.35	−1.95	2.64	0.77
**300 m**	76.57	68,303	1.02	0.99	1.06	0.21	0.22	−1.67	2.11	0.82
**Distance to the nearest road (b)**										
**Freeways**	1766.80	68,303	1.04	0.96	1.14	0.35	−7.80	−12.50	−3.09	< 0.01
**Major roads**	253.05	68,303	0.94	0.87	1.01	0.07	−1.95	−5.42	1.51	0.27

a) Adjusted for maternal age, length of gestation and poverty using smoothing splines and maternal race/ethnicity, insurance, parity, and gender of the infant as categorical variables.

b) The units are parts per million for CO, parts per billion for NO, NO_2_, NO_x_, and O_3_, and μg.m^-3^ for PM_10_ and PM_2.5_. Concentrations are averages, across the pregnancy period, derived from daily 24 h- mean concentrations for NO_2_, NO, NO_x_, CO, PM_10_ and PM_2.5_ and from daily mean concentrations from 10 am to 6 pm for O_3_. The unit for traffic density is vehicle number per day/meter. The unit for distance to road is meters.

When term birth weight is analyzed as a continuous variable, increases in mean birth weight are associated with most air pollution metrics (Table 3). This is notably the case when most monitoring station measurements (for NO_2_, NO, NO_x_, CO, PM_2.5_ and PM_10_, but not O_3_) are considered as air pollution metrics (range in point estimates: 20–36 g weight gain per inter-quartile range increase in pollutant concentrations). Slightly lower increases in mean birth weight are observed when predictions from the LUR (NO_2_ and NO_x_) or CALINE4 (NO_x_, CO and PM_2.5_) models are considered (range of point estimates: 6–16.5 g per inter-quartile range increase in pollutant concentrations). No statistically significant change in mean birth weight is associated with traffic density or proximity to the nearest main road, whereas a significant decrease is associated with a higher distance between maternal home and the nearest freeway. Conversely, a significant decrease in mean birth weight is associated with O_3_ ambient concentrations as measured by ambient monitoring stations (point estimate of 31 g weight loss per inter-quartile range increase in concentrations).

These results are robust to adjustment for maternal covariates which were not introduced in the main models (e.g., pregnancy conditions, maternal weight gain during pregnancy) or for census block group level variables other than poverty (population density, median income). However, after further adjustment of the main model for hospital or county, the increased risk of LBW associated with O_3_ is no longer significant, whereas the odds ratio for the proximity of the nearest major road is significant at the 5% level [see Additional file 3]. Adjusting for time (month and year) of conception has modest impacts on results. However when monitoring station measurements are considered, this yields a significant decrease in risk associated with PM_2.5_, while the initially marginally significant decrease in risk associated with NO_x_ disappears [see Additional file 3]. Adjusting for time of conception also cancels out the increases in mean birth weight associated with LUR estimates (NO_2_ and NO_x_). The impact of an adjustment for time of conception is not clear though, since this may cause over-adjustment when time-varying air pollution metrics (such as monitoring station measurements) are considered. Replicating the main analyses without adjusting for gestational age had no noteworthy impact on the results [see Additional file 4].

Analyses focusing on monitoring station measurements averaged by trimester do not produce markedly different results from those based on indicators averaged over the whole pregnancy. The only consistent pattern is that, for all pollutants except O_3_, odds ratios for LBW are lower and/or increases in birth weight are stronger in response to the pollutant concentrations experienced during the second trimester. A reverse pattern is observed for O_3_: the second trimester shows the highest, and the only significant, increase in risk of LBW [see Additional file 5].

## Discussion

In this hospital-based study of more than 70,000 term births, we observed contrasted results according to the different air pollution metrics examined. The risk of LBW is positively associated with ambient O_3_ concentrations but negatively associated with NO_2_, as measured by monitoring stations. LBW risk is also positively associated with traffic density and proximity to major roadways, while no significant association is observed for other air pollution metrics. When birth weight is analyzed as a continuous variable, increases in mean birth weight are associated with most air pollution metrics. However, no such increase is observed for indicators of traffic density or proximity to major roadways, and a significant decrease in mean birth weight is associated with ambient O_3_ concentrations.

Each of the air pollution metrics that we used has respective virtues and limitations. Measurements from monitoring stations are available over large regions and effectively reflect temporal variations in the mean ambient pollution level at each monitoring site, but their lack of geographical resolution is a serious limitation for pollutants with fine scale spatial variation such as NO_x_ and CO. Limited resolution might be less critical for pollutants characterized by strong regional components such as PM_2.5_. But still, the composition of particulates and their possible effects on intrauterine growth might be influenced by local sources, and thus vary at a small spatial scale [22]. The LUR model predictions that we used, captured small scale spatial contrasts in NO_2_ and NO_x_ concentrations effectively. However, the assumption that small scale spatial contrasts in NO_2_ and NO_x_ concentration remained stable throughout the study period (i.e., the assumption that at each residential address, the temporal variability of ambient concentrations was identical to that observed at the nearest monitoring site) might have been inappropriate [13]. Since the LUR models were developed using measurements for more recent years (2006–2007), there are uncertainties regarding their extrapolation backward in time [23].

Measurements from monitoring stations as well as predictions from LUR models do not reflect the sole influence of local traffic as a source of air pollution. They also integrate some regional contributions from traffic and from residential emissions. Conversely, predictions from the CALINE4 model specifically capture spatial contrasts in the dispersion of primary pollutants emitted by local traffic (on roadway segments up to 3 km from maternal homes). These predictions integrate the influence of meteorology and temporal variability in emissions, though for the latter at a coarser temporal resolution than do monitoring station measurements, because of the limited temporal resolution of input data (e.g., annual average traffic flow). Since traffic flow data are also more accurate for State highways than for major roads in California because of different assessment methods [24], we suspect that our CALINE4 predictions are more accurate for freeways and highways than for major roads. This would explain why CALINE4 predictions are moderately negatively correlated with distance to freeways, whereas they are weakly correlated with distance to major roads (see Table 2).

Air pollution is a mix of a very large number of components [9,25,26] and we could only measure or model a fraction of criteria pollutants which are monitored for regulatory purposes. We cannot discard the possibility that some individual air pollutants (or mix of these), which in our study setting were not strongly correlated in space or time with the criteria pollutants we focused on, might impair intrauterine growth. Considering these uncertainties, our findings based on simple indicators of traffic density and distance to major roadways are thought provoking. These indicators are arguably rather crude proxies for emission sources of traffic-related pollutants [27] and may result in substantial exposure misclassification, although not necessarily in a way that would differentially affect cases and controls. Their main strengths are the integration of a spatial dimension at a very fine scale (here for traffic density, a few hundred meters or less), and their relative specificity with regard to an identified source of air pollutants. They might thus capture, though imperfectly, the effects of some primary emissions of traffic-related pollutants, or mixes of such pollutants, which were neither measured nor modeled in our study.

While traffic density within a few hundred meters from maternal homes and proximity to the nearest main road are associated with increased risk of LBW but not with changes in mean birth weight, a statistically significant decrease in mean birth weight is associated with a longer distance between maternal home and the nearest freeway. Nevertheless, this result may be sensitive to the distribution of distances. Traffic-related pollutants decay to background concentrations by 160–570 m from the edge of roadways during daytime [28], and up to 2500 m downwind from freeways before sunrise [29]. However even under the latter condition (night time with more stagnant air), concentrations decline more sharply within 1000 m from freeways than at more remote distances [29]. This is likely because of the increased relative contribution of other sources (e.g. surface streets) to the pollutant concentrations at locations further away from a freeway. Therefore, the signal from freeway emissions appears to be more reasonably captured within 500 or 1000 m from freeways than at more remote distances. When analyses are restricted to subjects living no farther than 1000 m from a freeway (36% of the subjects, whereas 98% of the subjects were living within 1000 m of a main road), mean birth weight might slightly increases as distance from the nearest freeway increases, although this result is not statistically significant [see Additional file 6].

All of our air pollution metrics share limitations that are still common in the field of air pollution epidemiology and birth outcomes [12,22]. The personal exposure of mothers during pregnancy, that is hypothesized to influence birth weight, could not be estimated in this large cohort since we could not take into account specific time-activity patterns [17] and ambient pollutant concentrations prevailing in various living micro-environments such as the workplace or public transportation. The ignorance of these factors undoubtedly contributed to exposure measurement errors, of which direction and magnitude might differ depending on the air pollution metrics used [30]. This makes the comparison of results according to different air pollution metrics difficult, since each of them is probably not related to personal exposure in the same way. In spite of a few published studies on the topic [31,32], the relationships between personal exposure of pregnant women and the pollution metrics that we used would warrant more research in the future. In addition, our air pollution metrics relied on maternal home address at the time of delivery. For mothers who moved during pregnancy, we are unaware of the locations of other homes occupied before delivery. All these sources of exposure measurement error contribute random error to the epidemiologic results, and might also potentially generate bias.

The observed increases in mean birth weight associated with most air pollution metrics were unexpected. Since many statistical tests were applied as part of this study, with a 5% type I error risk for each, some of these significant associations might be pure chance findings. This is not likely to explain all of the significant associations we observed, however: out of the 246 statistical tests conducted, 43% were statistically significant. Although some air pollutants like diesel exhausts and polycyclic aromatic hydrocarbon have been identified as endocrine disruptor compounds [33,34], which are increasingly suspected to play a role in childhood and adulthood obesity [34,35], there is, to the best of our knowledge, no proposed biological mechanisms by which endocrine disruptors would cause increased fetal weight gain. The possible induction of gestational or preexisting diabetes by air pollution (which is a current research question [36,37]) might have constituted another plausible explanation for such results since both conditions may cause increased birth weight. However, our results were unaffected by adjustment for maternal diabetes. Last, a “harvest” effect of increased miscarriage among the most susceptible fetuses (which would have been more likely to have lower weights at birth) might hypothetically be triggered by exposure to air pollution and result in increased mean birth weight associated with this exposure. However, it is difficult to evaluate that hypothesis without data on miscarriages. We are not aware of any other hypothesized mechanisms for a causal relationship between exposure to air pollution and increased birth weight.

Lack of adjustment for one or several unmeasured confounders is a possible explanation for such findings. We controlled for a large set of individual potential confounders, including race/ethnicity, insurance status and maternal conditions, as well as neighborhood socioeconomic factors. Our results are robust to adjustment for further available covariates. However, we had no direct information on the height of the parents, the body mass index of mothers at the beginning of pregnancy (both usually positively associated with birth weight [38]) or their smoking habits (usually negatively associated with birth weight [39]). Adjustment for insurance status, poverty (two proxy variables for socioeconomic status) and race/ethnicity should have contributed to partially adjust for maternal smoking and pre-pregnancy body mass index. However, the possibility of residual confounding by these factors, and possibly by others (traffic-related noise, residual differences in socioeconomic status not reflected by insurance status and neighborhood-level variables, and maybe unknown factors), still remains.

If some confounding factors are truly responsible for the observed increases in mean birth weight associated with most air pollution metrics, the fact that no increase in mean birth weight is associated with local traffic density (within a few hundred meters from maternal homes) or distance to main roads might reflect different possible situations. This might indicate that the different air pollution metrics that we studied are associated in various ways with potential confounding factors. Alternatively, if all our air pollution metrics are similarly associated with potential confounding factors that bias the results “upward” (i.e., toward observed increases in birth weight), the fact that no increase in birth weight is associated with traffic density or distance to main roads might mean that they indeed exert an effect “downwards” (i.e., generate decreases in birth weight). Since these two possibilities cannot be disentangled without measurement of additional variables, the contrasting results suggests the importance of further birth weight investigations that measure air pollutants, noise, and other confounders potentially associated with local traffic.

Comparing our results to those of previous studies is not straightforward, in part because of differences in the natures and definitions of the air pollution metrics employed, varying birth outcomes of interest, as well as available information on potential confounders and strategies employed for statistical analyses. However, two recent reviews and meta-analyses attempted to summarize the findings of studies focusing on the relations between birth weight and exposure to criteria pollutants [10,22]. Overall, our findings of increases in mean birth weight associated with ambient air pollution concentrations measured by monitoring stations disagree with those of most published studies [10,11], including those conducted in California [25,40]. A selection effect might account for such differences. Compared to previous studies conducted in California [40] and Los Angeles County [25] that used birth certificates during similar study periods, our hospital-based cohort has lower percentages of Hispanic mothers (32% versus 50% [40] and 70% [25]) and of mothers relying on public insurance (28% versus 66% [25]). Our cohort also has a higher percentage of Caucasian mothers (41% versus 30% [40] and 13% [25]). The percentage of term LBW infants is also lower in our cohort (1.7%) than in the California (2.3%) [40] and Los Angeles County (2.1%) studies [25]. Similar differences are observed when our cohort is directly compared to birth certificates from Los Angeles and Orange counties for year 2001 (that is, in the middle of our study period) [Additional file 7].

In a recent meta-analysis by Stieb et al. [10], ambient ozone concentrations were associated with a non-significant reduction in mean birth weight. Previous studies conducted in California reported significant decreases in mean birth weight associated with ozone concentrations [40,41], but a recent one found no association with LBW [25]. Results from the latest studies conducted in other settings are also mixed, ranging from significant decreases in birth weight [8] or increases in the risk of being small for gestational age [42] to reduced risk of LBW [26].

A rapidly growing number of studies have used predictions from LUR models for NO_x_, NO or NO_2_, with some results indicative of decreases in birth weight (e.g., [9,25]), while others are not significant (or mixed depending on the birth outcome considered) (e.g., [30]), or indicate an apparent increase in birth weight [43]. Only one used a line source dispersion model specific of traffic emissions, comparable to CALINE4 [44]. It reported a birth weight reduction associated with CO, in a selected sub-area of the study setting [44]. Traffic density indexes, with varying definitions, have been used in seven studies. Four of these reported decreases in birth weight [27,45-47], two reported null or mixed results [37,48] and one an apparent increase in birth weight [43]. Proximity to roadways was associated with decreases in birth weight in six studies [30,37,46,49-51], while two others reported no significant associations [43,48].

## Conclusions

This study shows a reduction in mean birth weight and an increase in the risk of LBW associated with ambient ozone concentrations, as well as increases in the risk of LBW associated with traffic density and proximity to roadways. For other commonly used air pollution metrics (ambient monitoring stations measurements for pollutants other than ozone, predictions from land use regression or Gaussian line dispersion model), we observed no increased risk of LBW and even some increases in mean birth weight, contrarily to most previously published studies. The potential influence of unmeasured confounding factors and/or measurement error cannot be discarded as possible explanations for these findings. Despite this, our results suggest that in our study population, ozone and primary traffic-related pollutants emitted in the proximity of roadways are more likely related to increased risks of LBW than the other dimensions of air pollution that we studied. Further studies are necessary to assess the consistency of these patterns across populations.

## Abbreviations

CO: Carbon monoxide (CO); IQR: Inter-quartile range; LBW: Low birth weight;LUR: Land use regression; NO: Nitric oxide; NOx: Nitrogen oxides; NO2: Nitrogen dioxide; O3: Ozone; PM10: Particulate matter of less than 10 μm in aerodynamic diameter; PM2.5: Particulate matter of less than 2.5 μm in aerodynamic diameter

## Competing interests

The authors declare that they have no competing interest.

## Authors’ contributions

OL conducted the statistical analyses and wrote the paper; JW developed the project, mentored the work, developed air pollution metrics and contributed to the writing of the paper; LL developed some air pollution metrics and critically reviewed the paper; JC was involved in the project development and critically reviewed the paper; SB provided advice on statistical analyses and critically reviewed the paper; All authors read and approved the final version of this manuscript.

## Supplementary Material

Additional file 1 Histograms of the distributions of air pollution indicators. Click here for file

Additional file 2 associations between mean birth weight and an inter-quartile range in air pollution metrics - restricted populations, for pregnancy-long air pollution metrics. Click here for file

Additional file 3 Sensitivity of the association between the air pollution metrics and birth outcomes to adjustment for hospital, county and time of conception. Click here for file

Additional file 4 associations between birth weight and an inter-quartile range in air pollution metrics, for pregnancy-long exposures, without adjustment for the length of gestation. Click here for file

Additional file 5 Associations between mean birth weight and an inter-quartile range in monitoring stations measurements, by pregnancy trimester. Click here for file

Additional file 6 Part A. Mean change in birth weight according to the distance to the nearest freeway (restricted to distances between 0 and 1000 m). Click here for file

Additional file 7 Descriptive statistics for term births in Los Angeles and Orange Counties birth certificates (2001) and in the Memorial Care database (1997-2006). Click here for file
