# Supplementary material for: Investigating the association between birth weight and complementary air pollution metrics: a cohort study
Source: Environ Health. 2013 Feb 17;12:18. doi: 10.1186/1476-069X-12-18 (PMC3599912; doi:10.1186/1476-069X-12-18)
Supplement: Additional file 1 — Histograms of the distributions of air pollution indicators. [file 1476-069X-12-18-S1.ppt]

## Slide 1
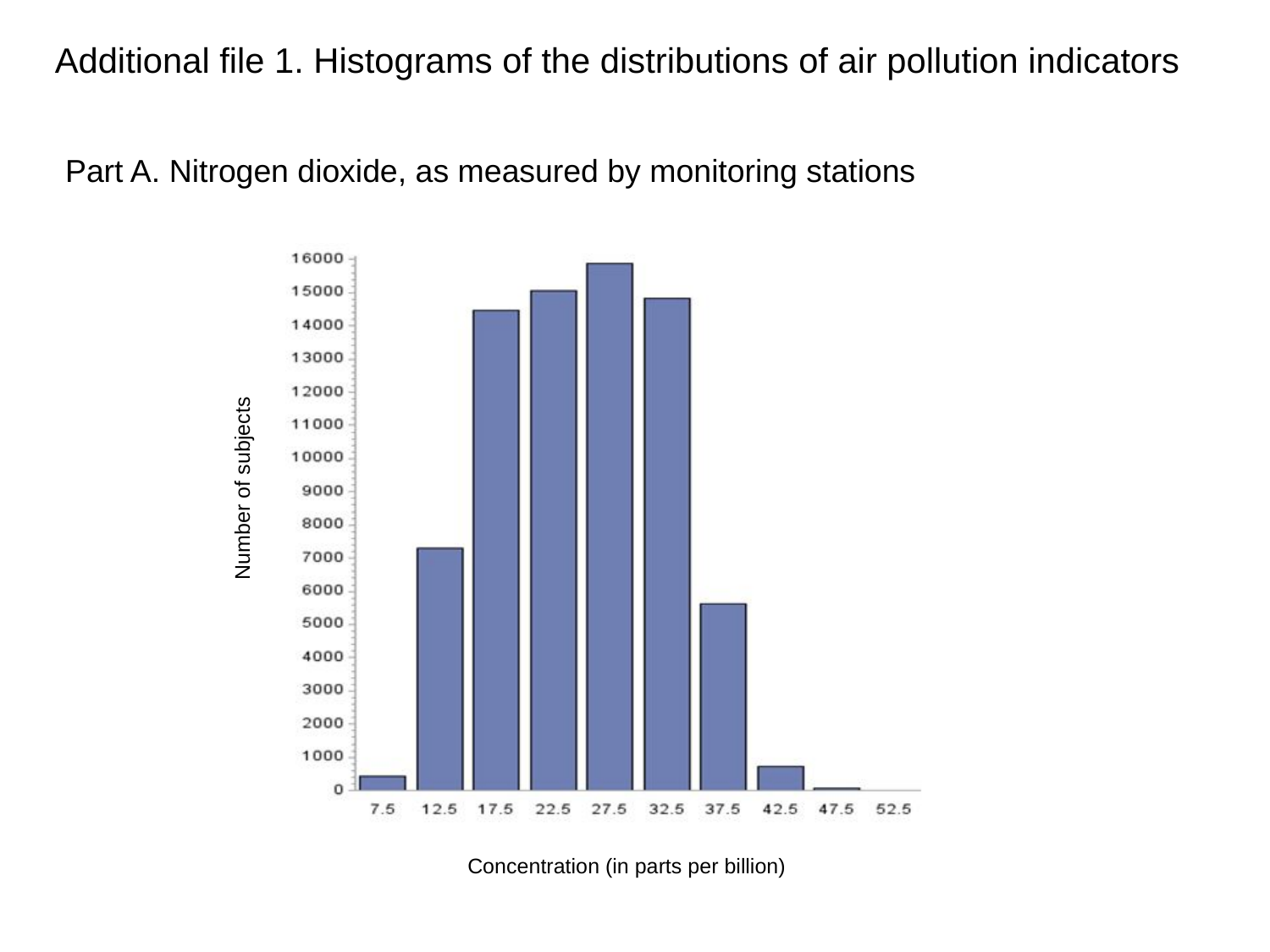

Additional file 1. Histograms of the distributions of air pollution indicators
Part A. Nitrogen dioxide, as measured by monitoring stations
Number of subjects
Concentration (in parts per billion)

## Slide 2
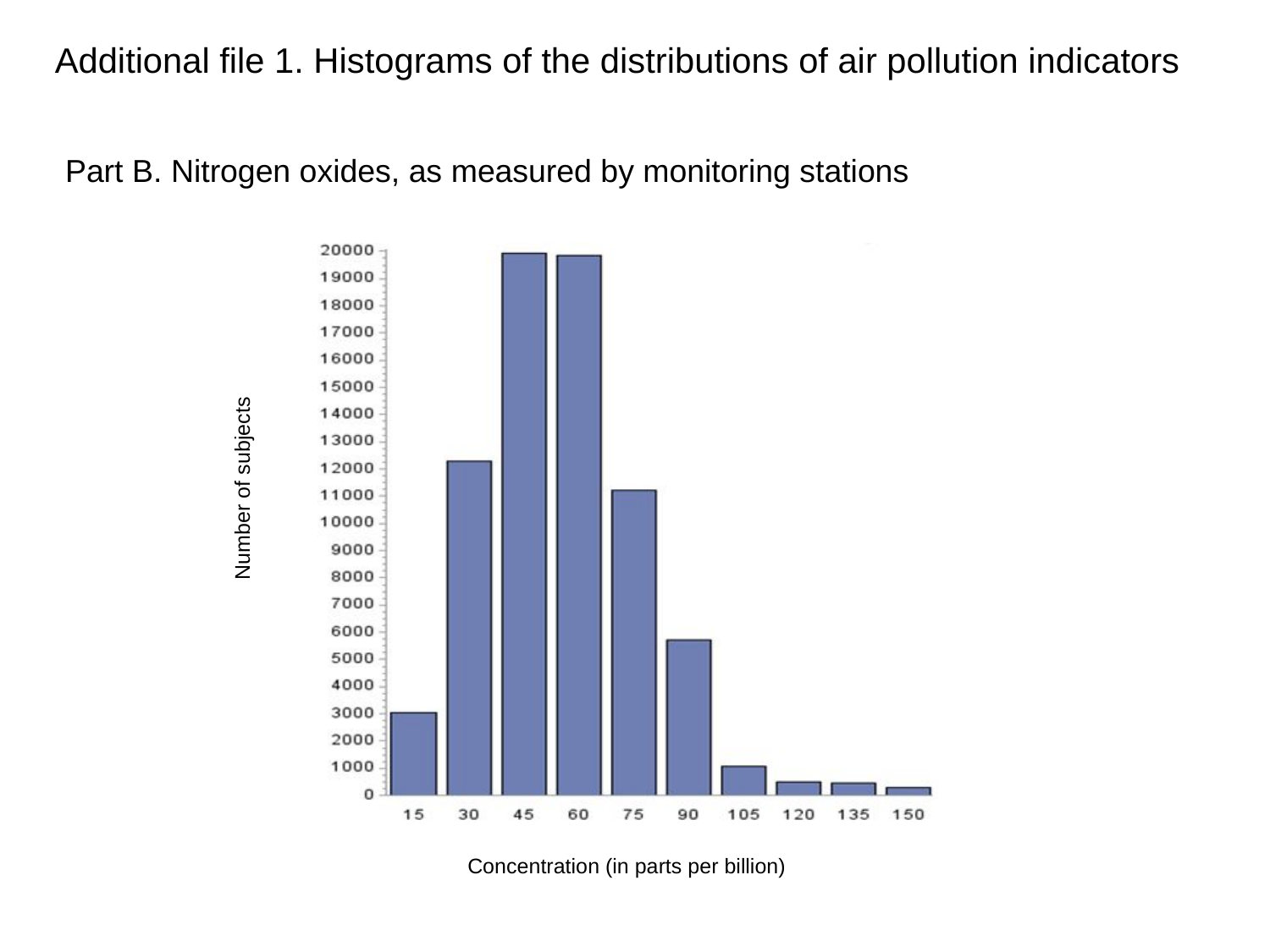

Additional file 1. Histograms of the distributions of air pollution indicators
Part B. Nitrogen oxides, as measured by monitoring stations
Number of subjects
Concentration (in parts per billion)

## Slide 3
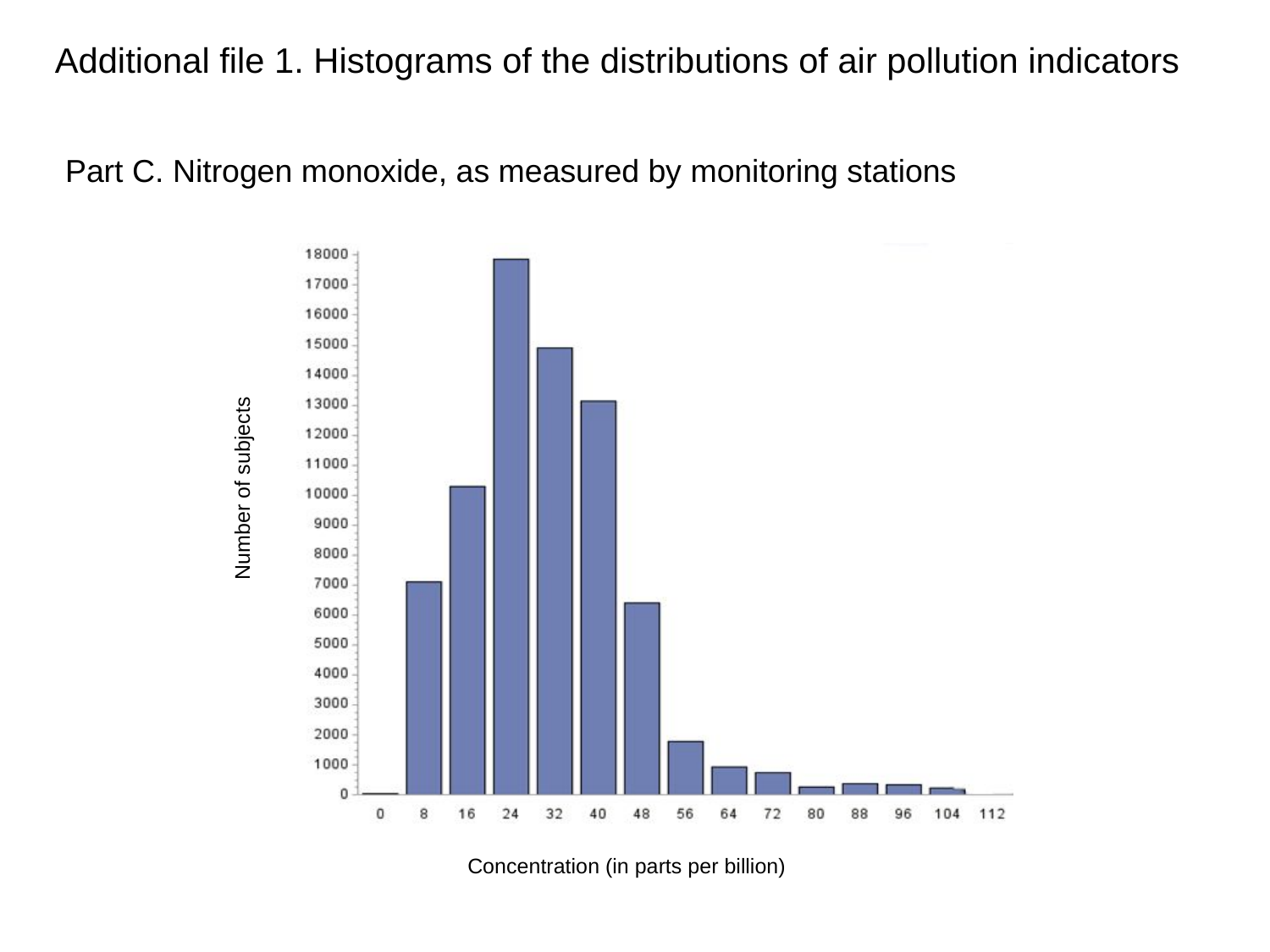

Additional file 1. Histograms of the distributions of air pollution indicators
Part C. Nitrogen monoxide, as measured by monitoring stations
Number of subjects
Concentration (in parts per billion)

## Slide 4
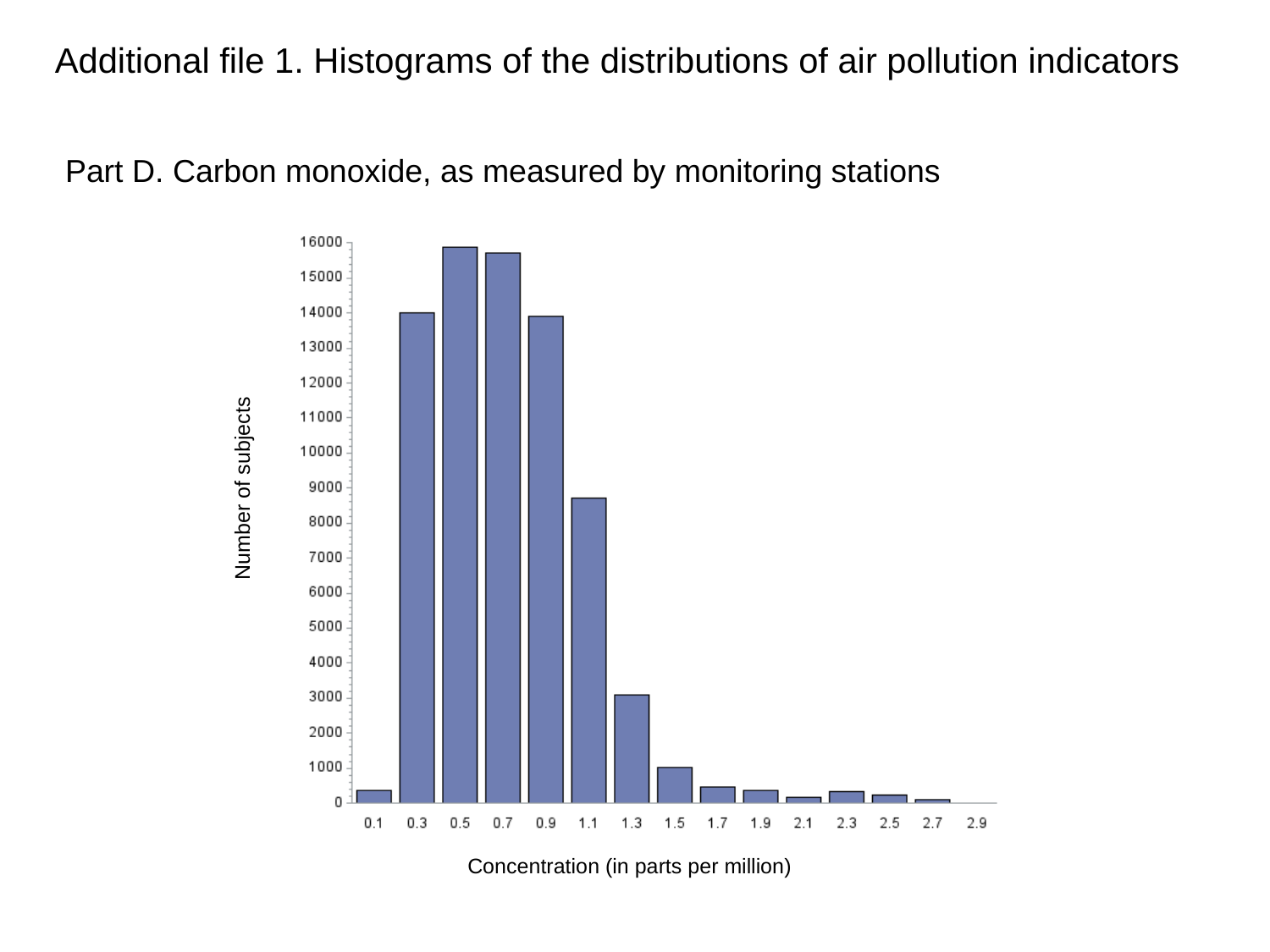

Additional file 1. Histograms of the distributions of air pollution indicators
Part D. Carbon monoxide, as measured by monitoring stations
Number of subjects
Concentration (in parts per million)

## Slide 5
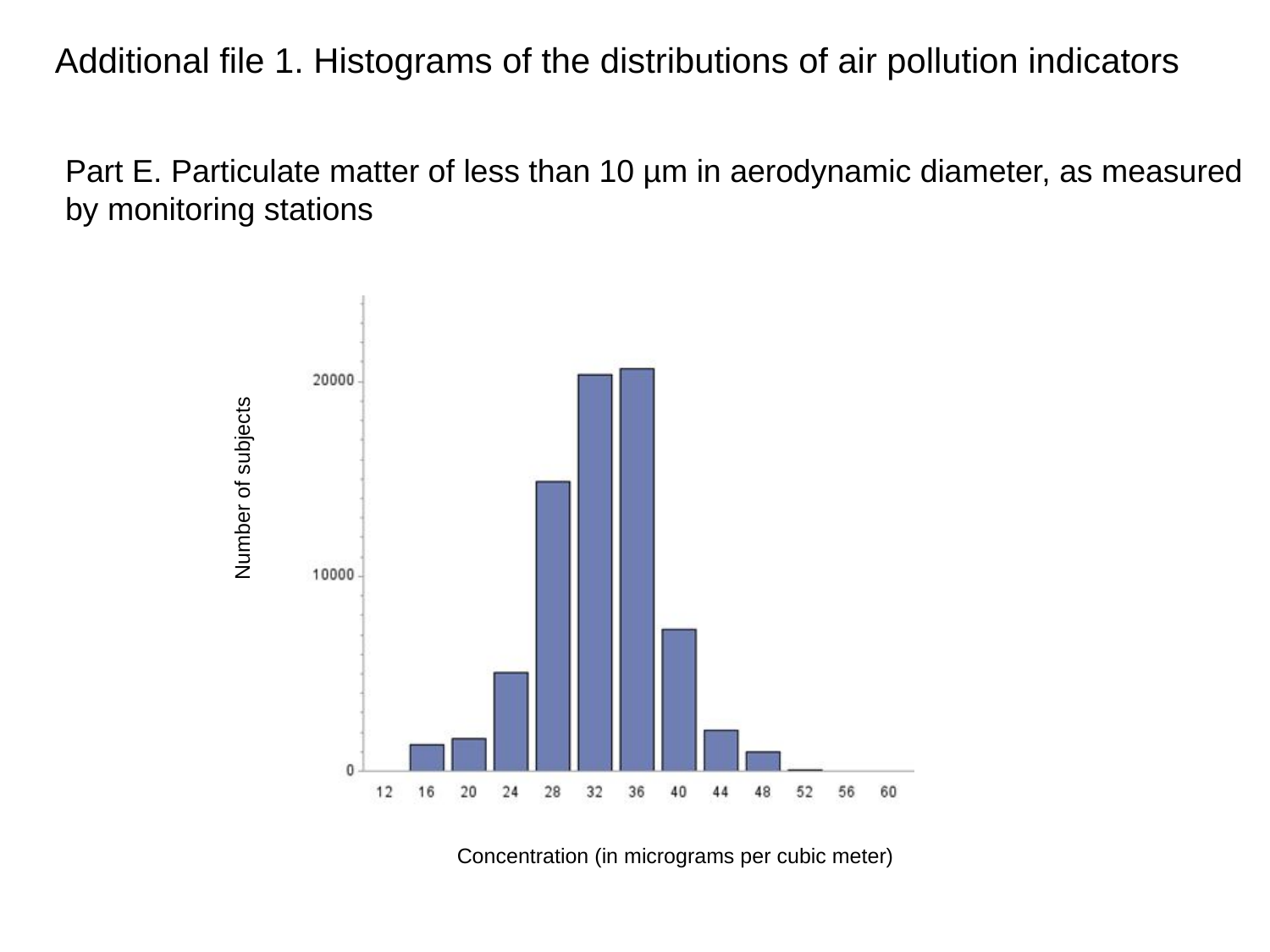

Additional file 1. Histograms of the distributions of air pollution indicators
Part E. Particulate matter of less than 10 µm in aerodynamic diameter, as measured by monitoring stations
Number of subjects
Concentration (in micrograms per cubic meter)

## Slide 6
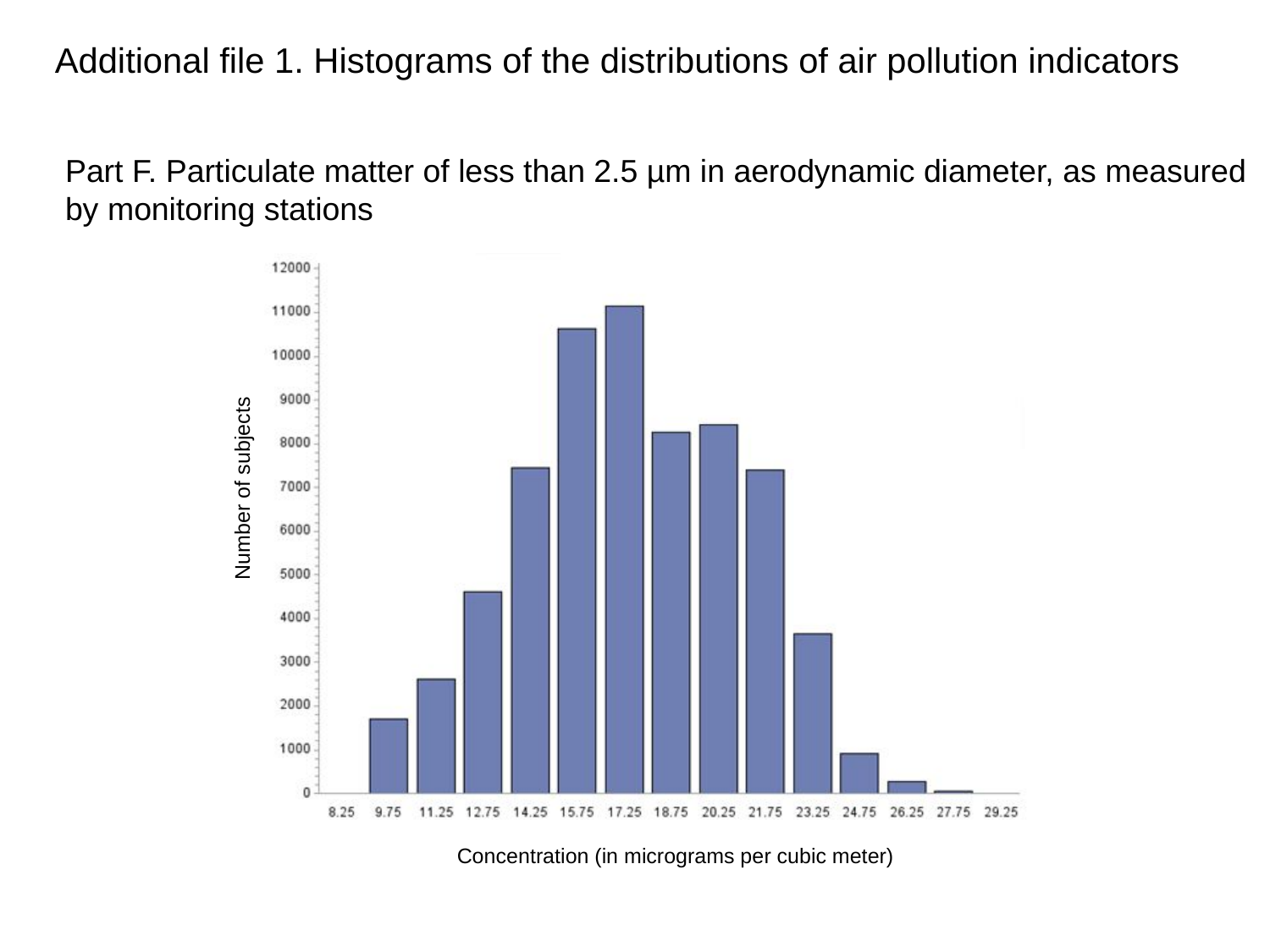

Additional file 1. Histograms of the distributions of air pollution indicators
Part F. Particulate matter of less than 2.5 µm in aerodynamic diameter, as measured by monitoring stations
Number of subjects
Concentration (in micrograms per cubic meter)

## Slide 7
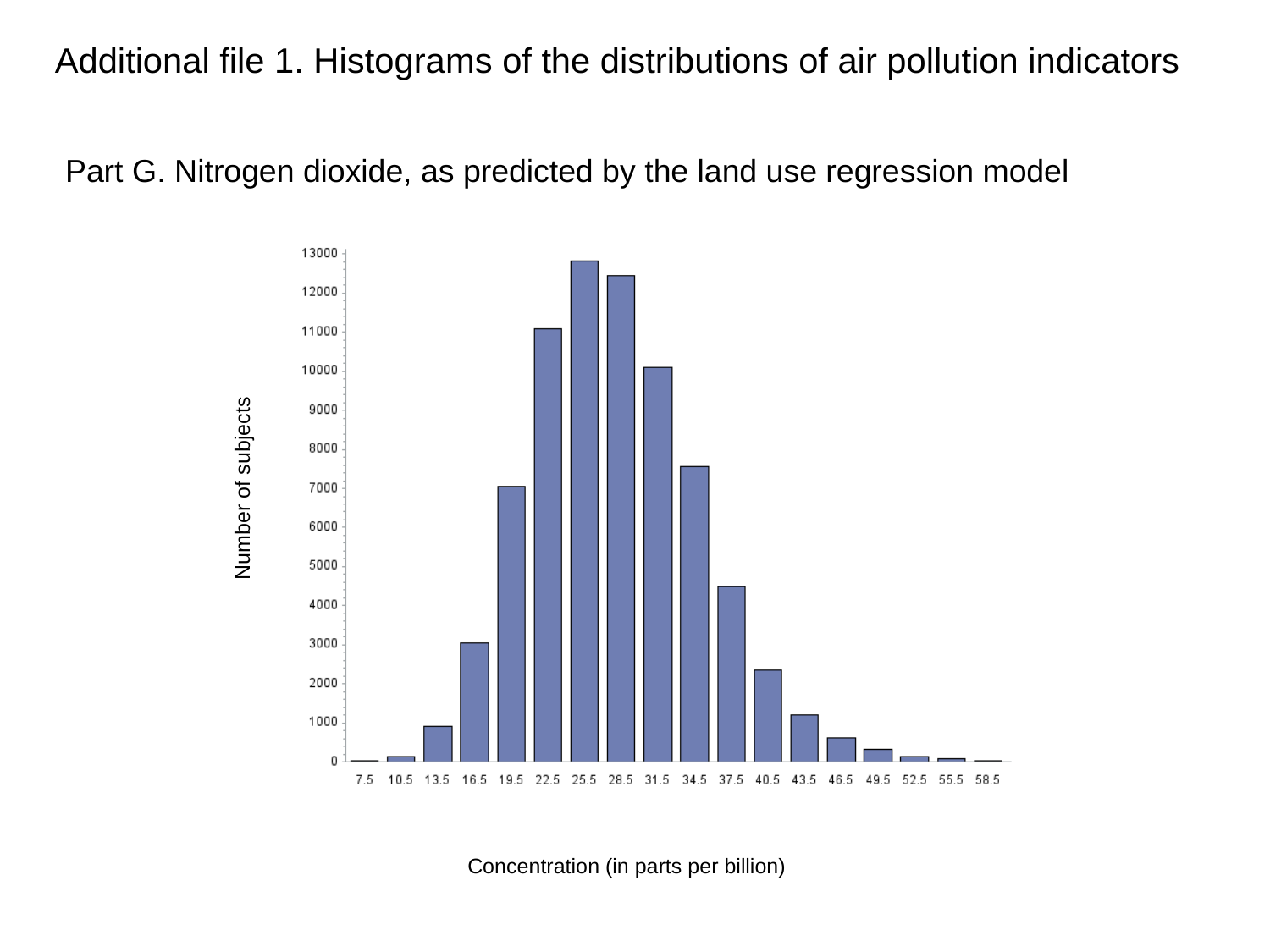

Additional file 1. Histograms of the distributions of air pollution indicators
Part G. Nitrogen dioxide, as predicted by the land use regression model
Number of subjects
Concentration (in parts per billion)

## Slide 8
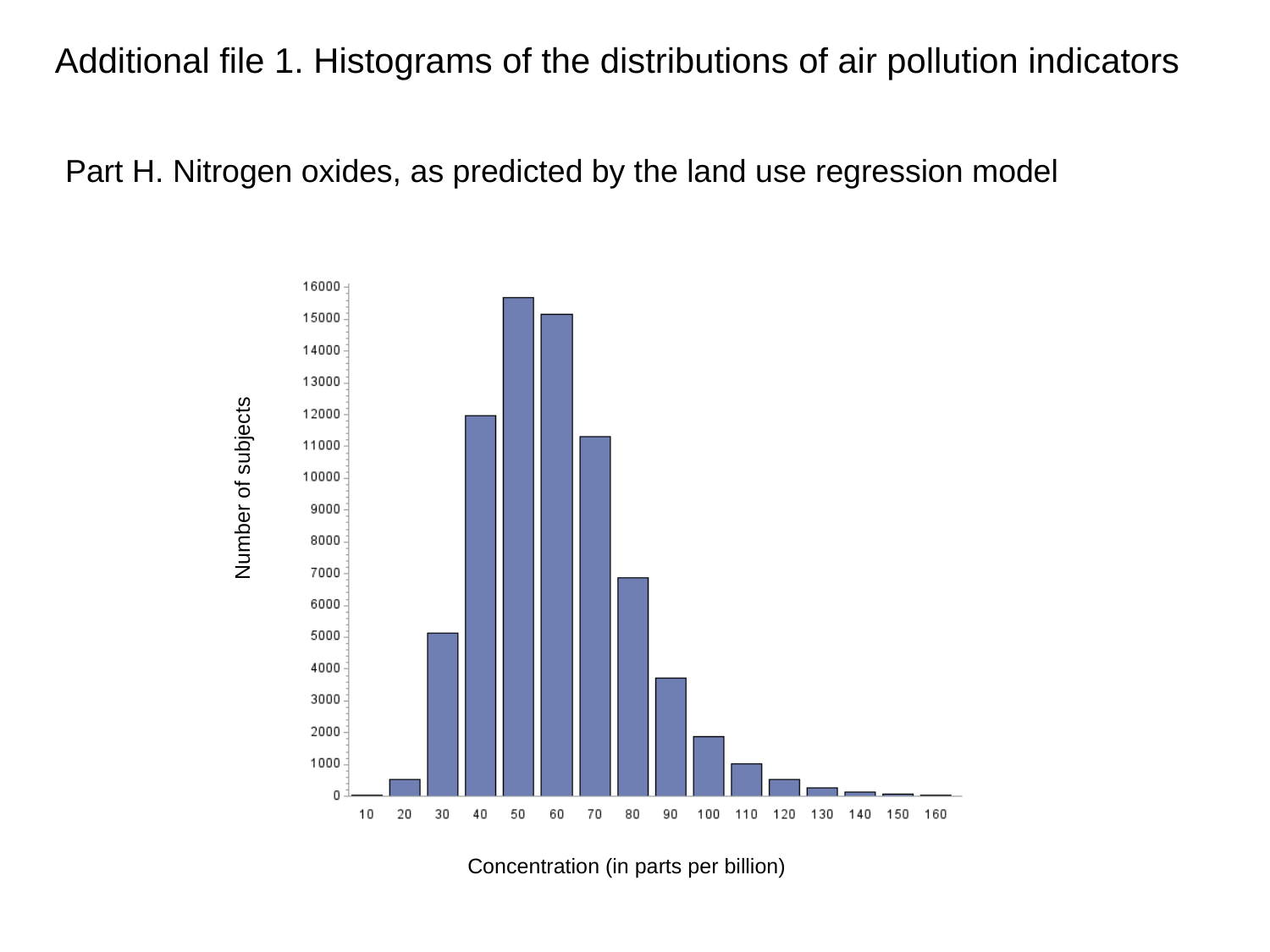

Additional file 1. Histograms of the distributions of air pollution indicators
Part H. Nitrogen oxides, as predicted by the land use regression model
Number of subjects
Concentration (in parts per billion)

## Slide 9
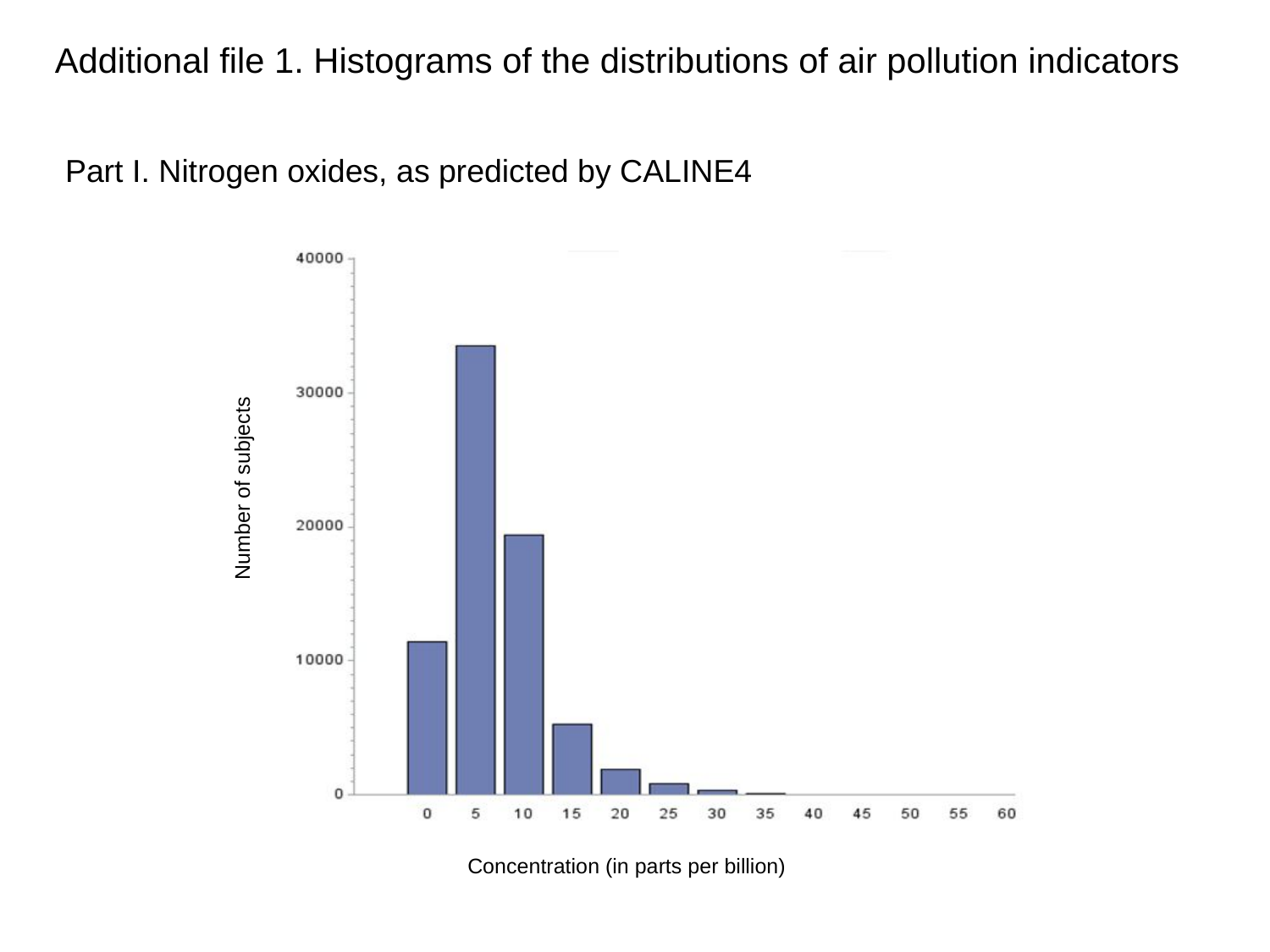

Additional file 1. Histograms of the distributions of air pollution indicators
Part I. Nitrogen oxides, as predicted by CALINE4
Number of subjects
Concentration (in parts per billion)

## Slide 10
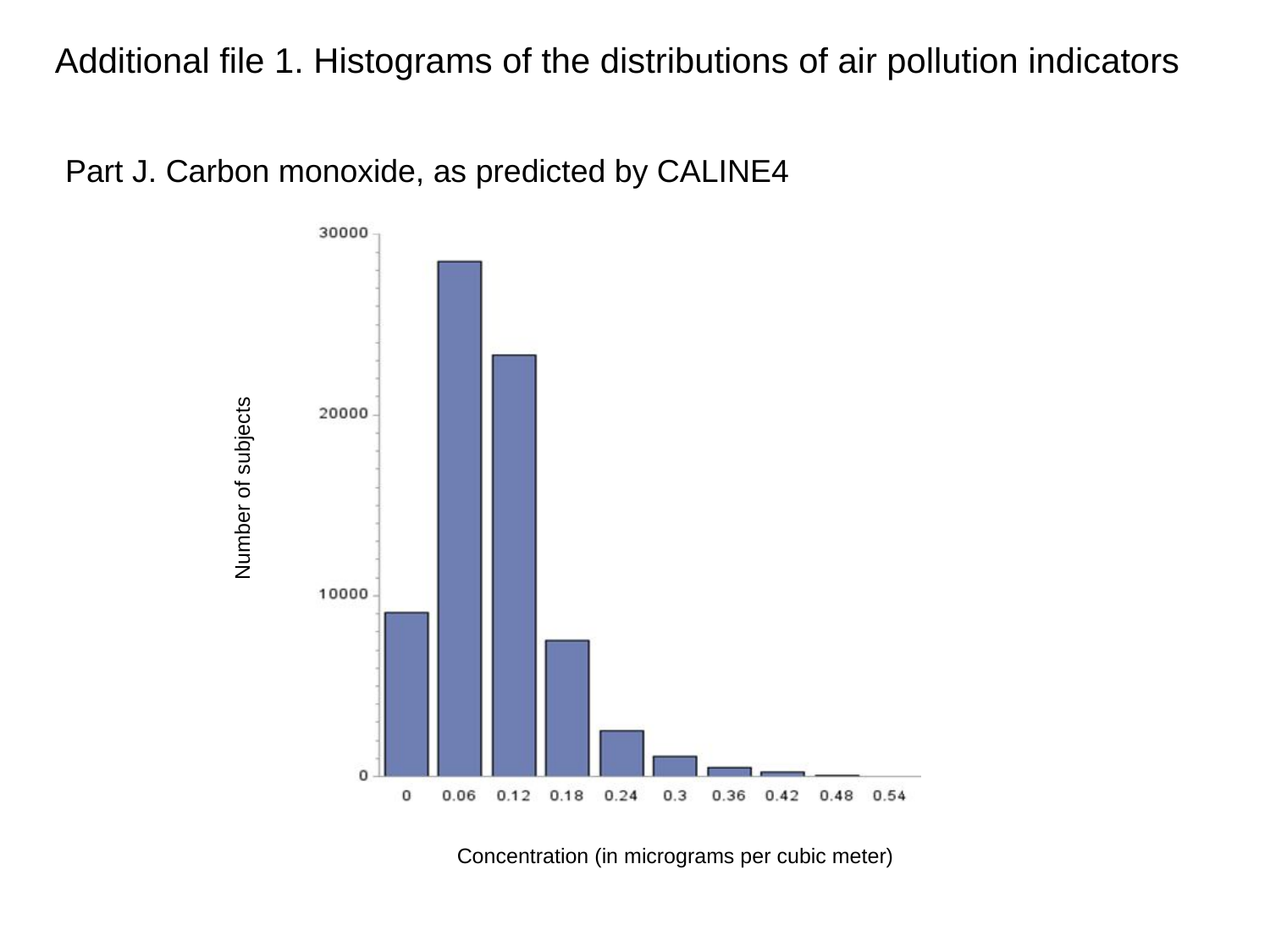

Additional file 1. Histograms of the distributions of air pollution indicators
Part J. Carbon monoxide, as predicted by CALINE4
Number of subjects
Concentration (in micrograms per cubic meter)

## Slide 11
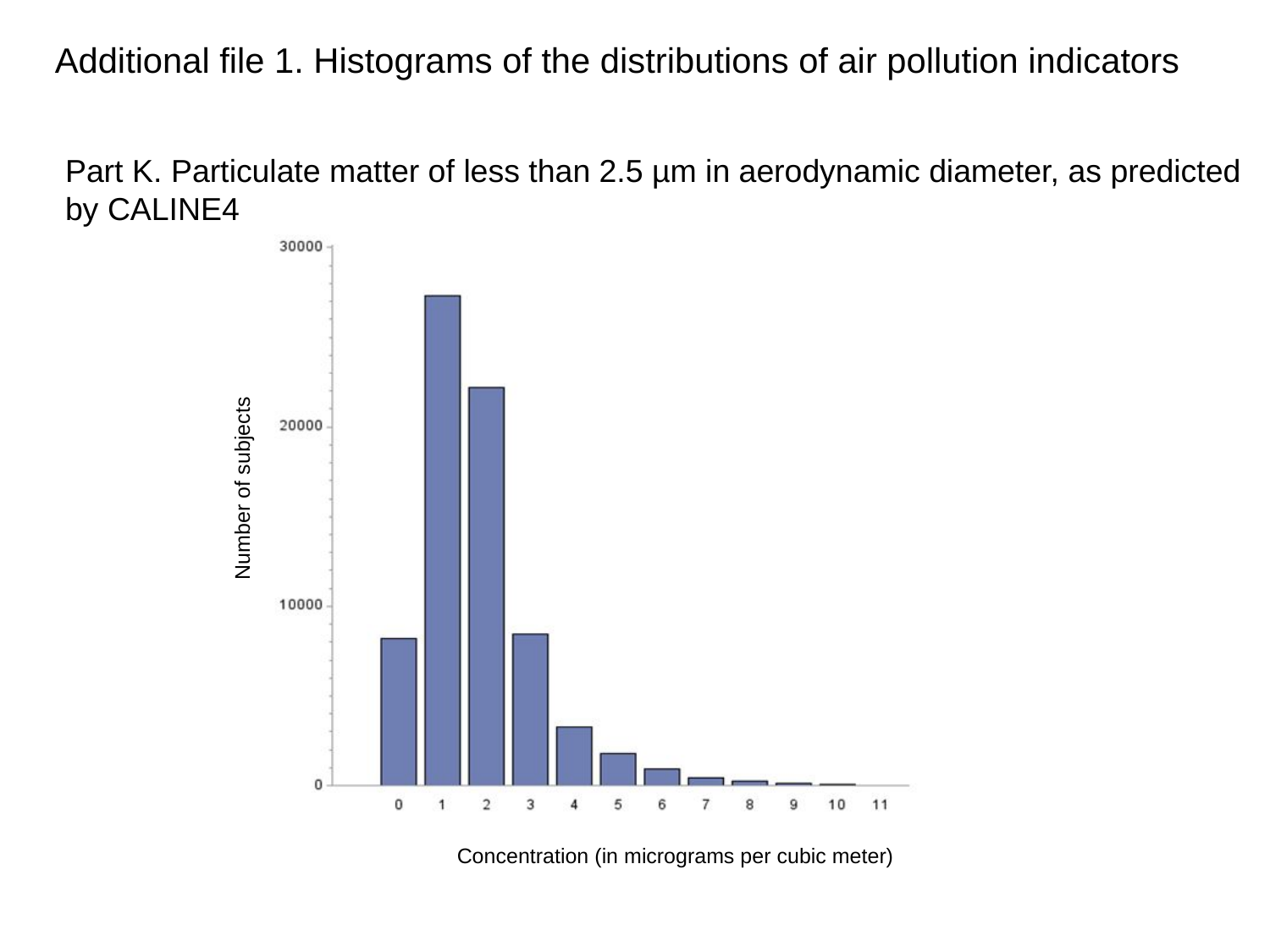

Additional file 1. Histograms of the distributions of air pollution indicators
Part K. Particulate matter of less than 2.5 µm in aerodynamic diameter, as predicted by CALINE4
Number of subjects
Concentration (in micrograms per cubic meter)

## Slide 12
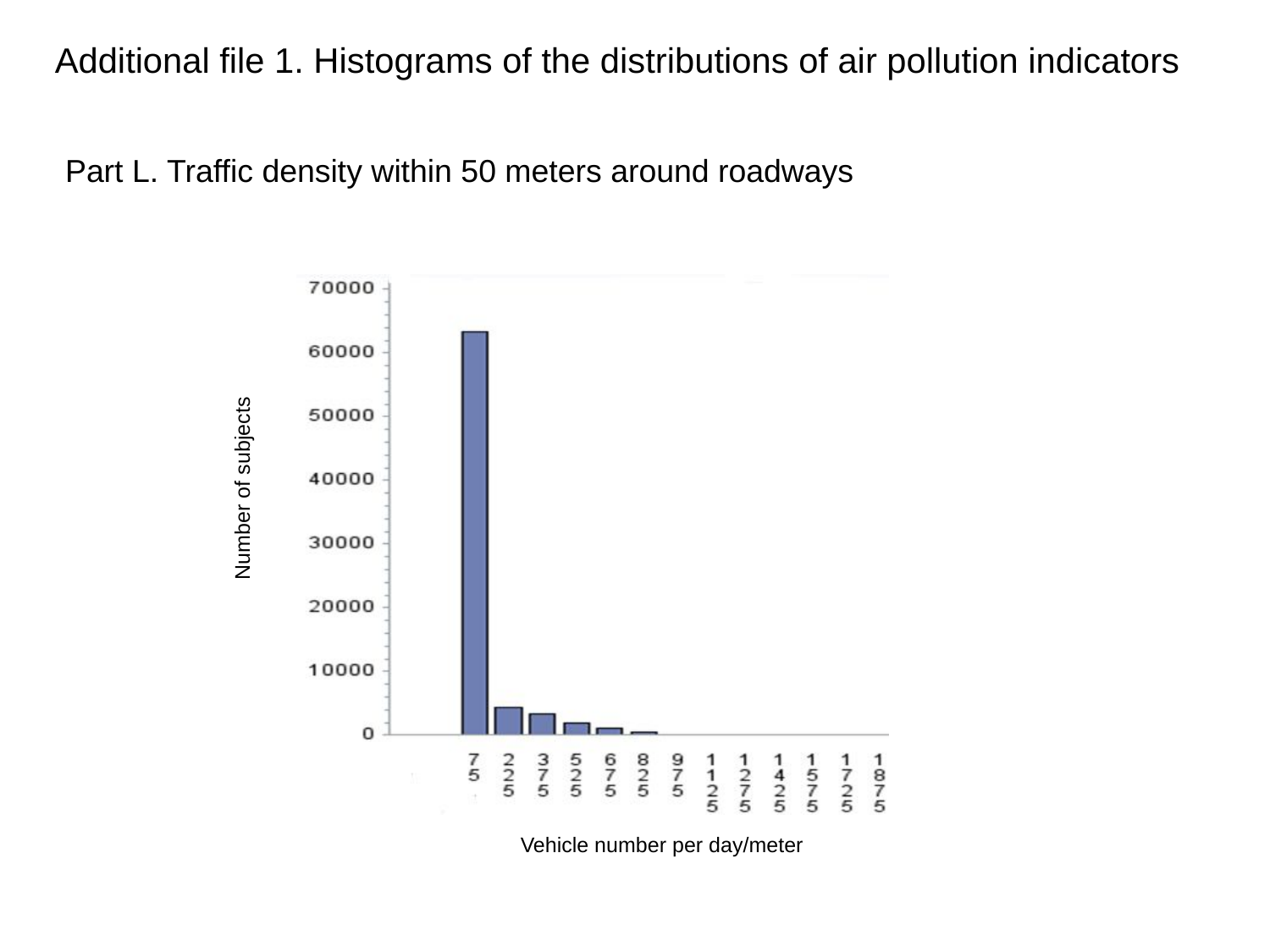

Additional file 1. Histograms of the distributions of air pollution indicators
Part L. Traffic density within 50 meters around roadways
Number of subjects
Vehicle number per day/meter

## Slide 13
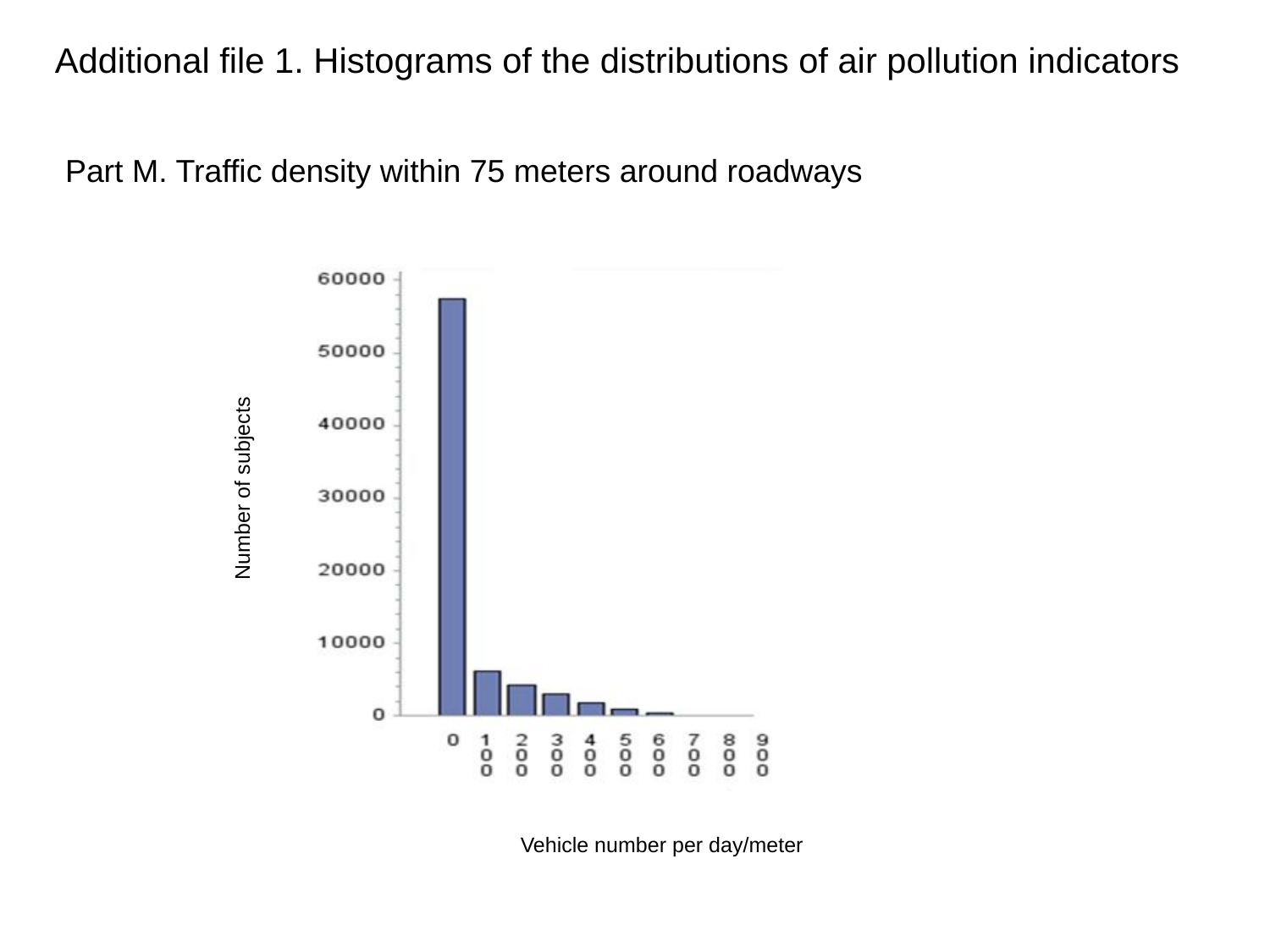

Additional file 1. Histograms of the distributions of air pollution indicators
Part M. Traffic density within 75 meters around roadways
Number of subjects
Vehicle number per day/meter

## Slide 14
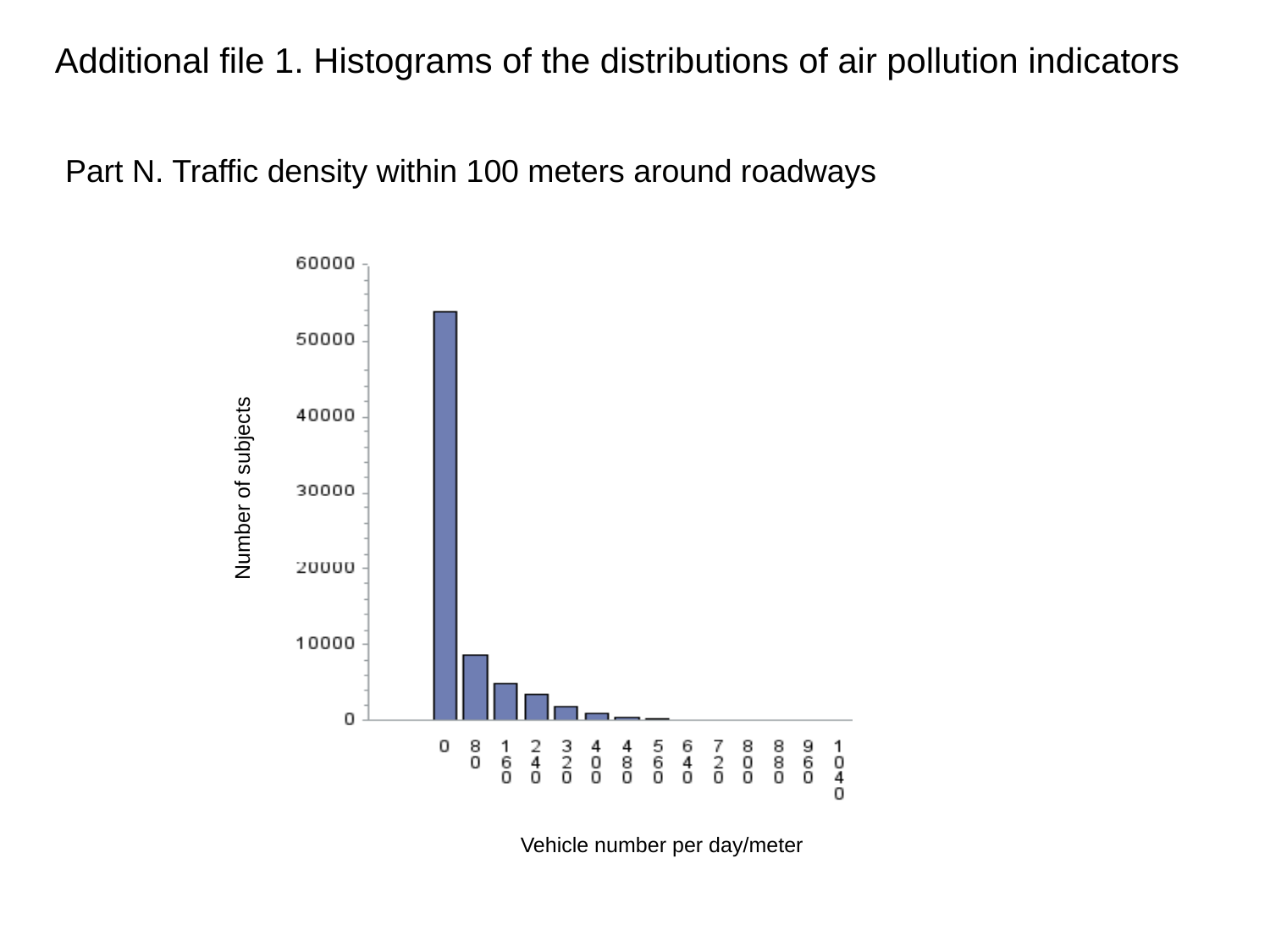

Additional file 1. Histograms of the distributions of air pollution indicators
Part N. Traffic density within 100 meters around roadways
Number of subjects
Vehicle number per day/meter

## Slide 15
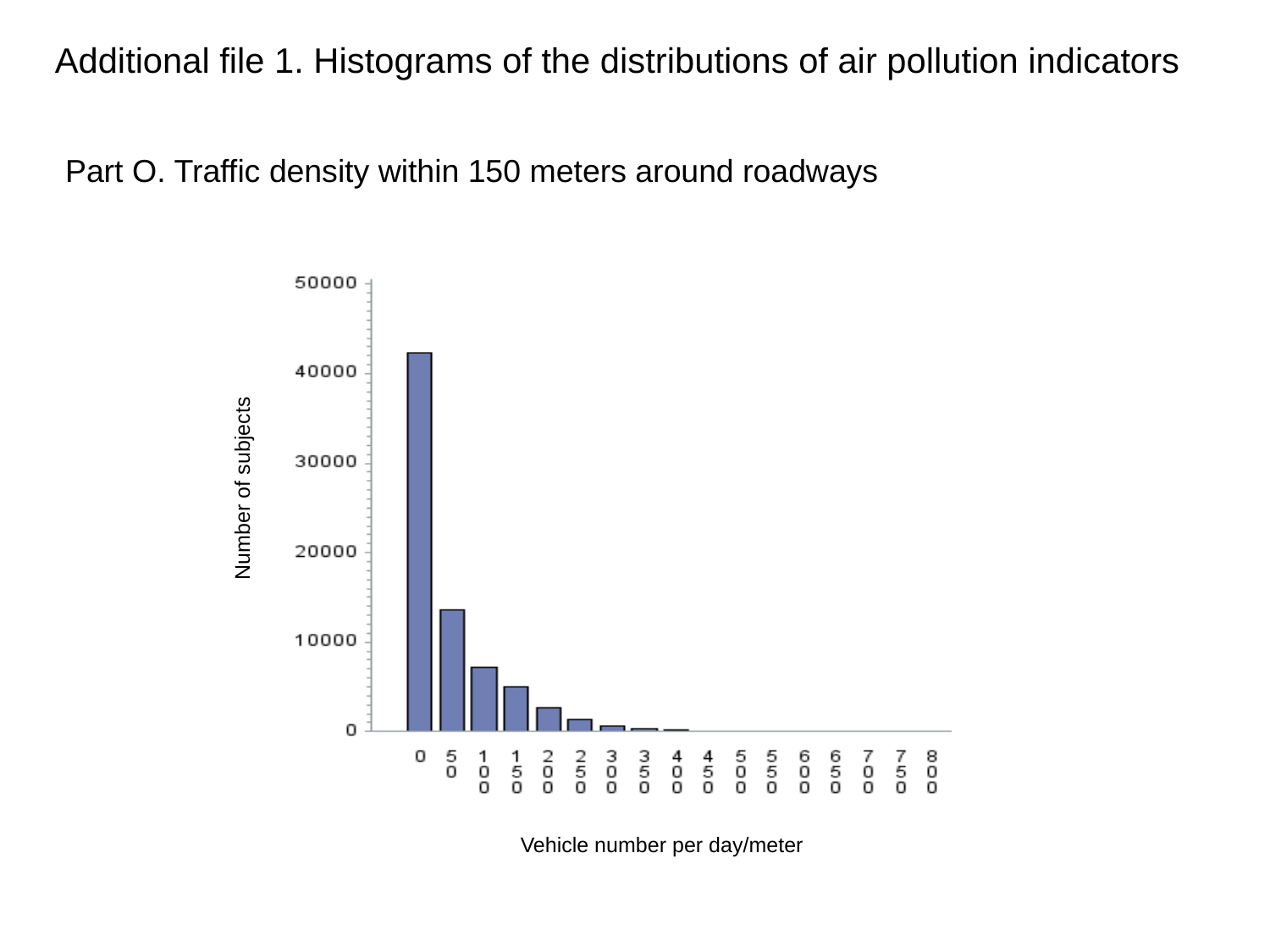

Additional file 1. Histograms of the distributions of air pollution indicators
Part O. Traffic density within 150 meters around roadways
Number of subjects
Vehicle number per day/meter

## Slide 16
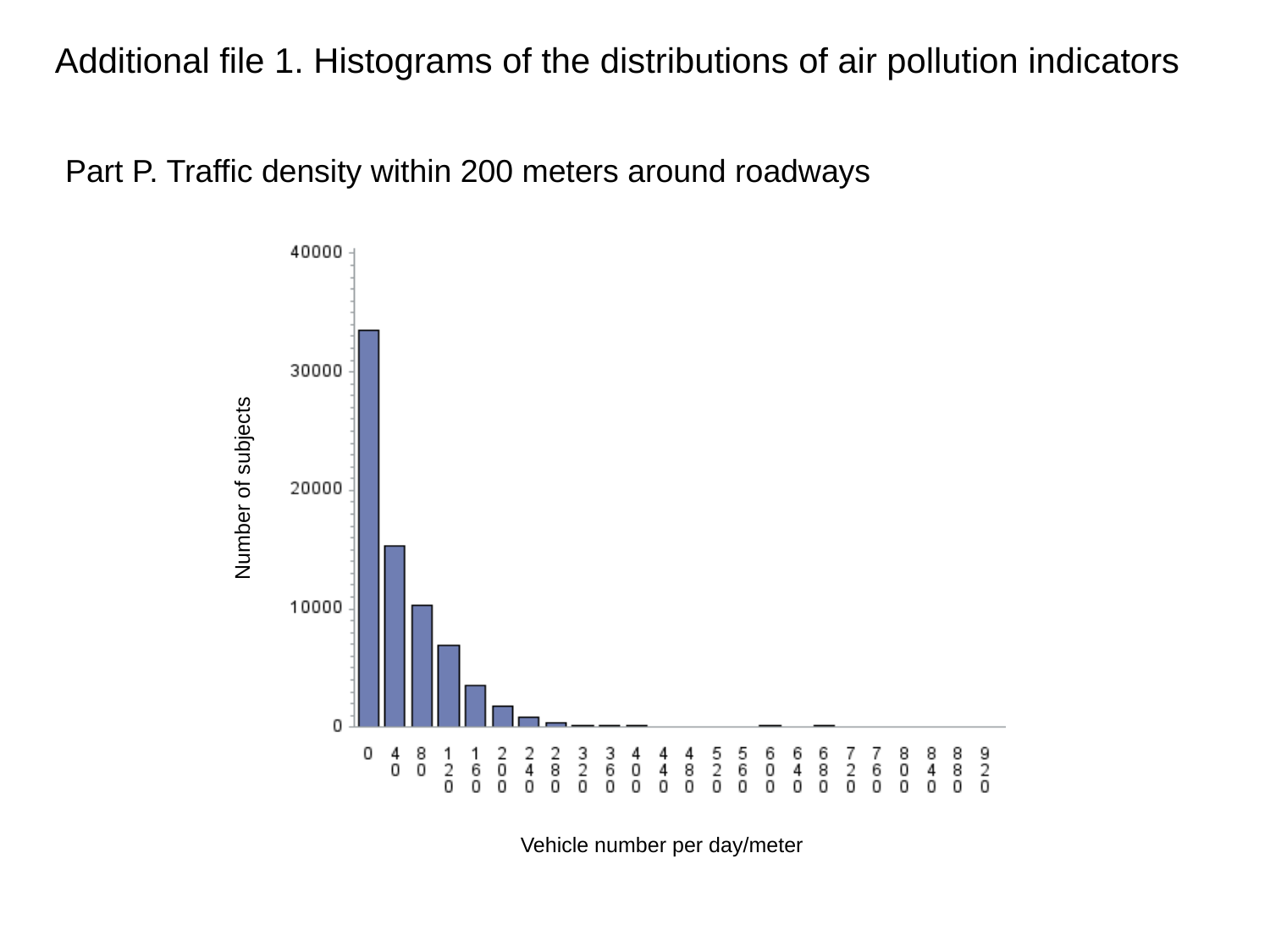

Additional file 1. Histograms of the distributions of air pollution indicators
Part P. Traffic density within 200 meters around roadways
Number of subjects
Vehicle number per day/meter

## Slide 17
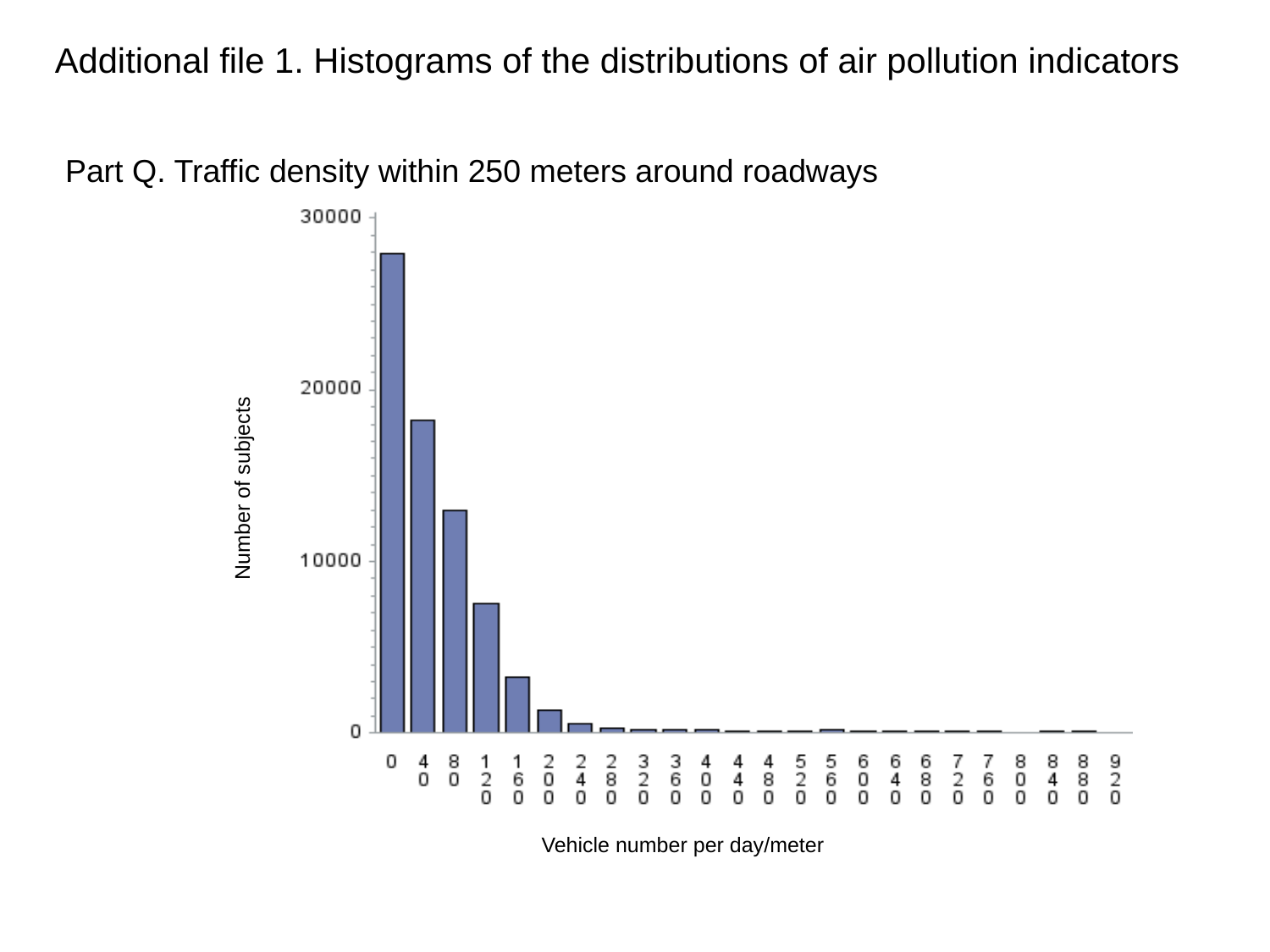

Additional file 1. Histograms of the distributions of air pollution indicators
Part Q. Traffic density within 250 meters around roadways
Number of subjects
Vehicle number per day/meter

## Slide 18
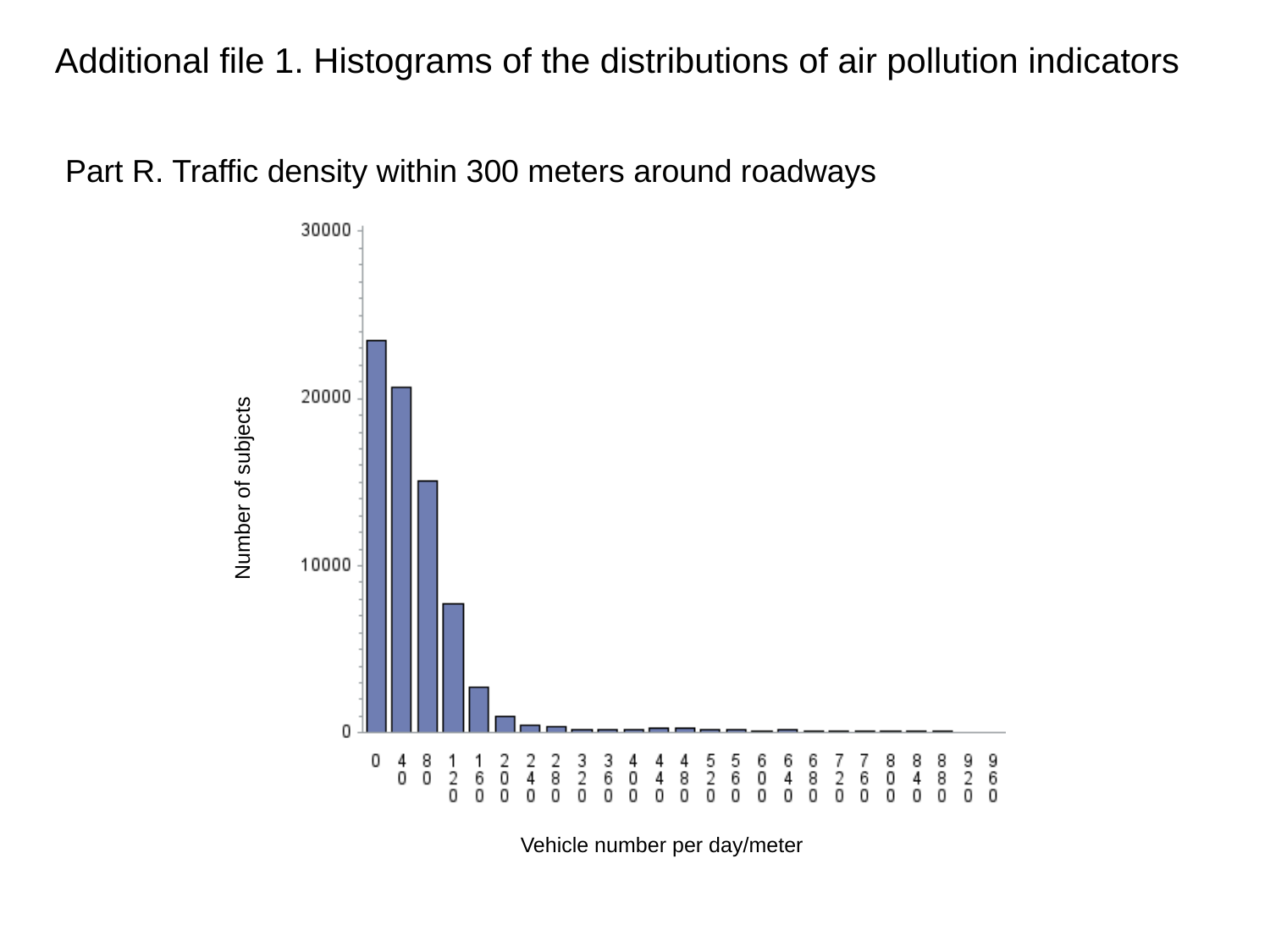

Additional file 1. Histograms of the distributions of air pollution indicators
Part R. Traffic density within 300 meters around roadways
Number of subjects
Vehicle number per day/meter

## Slide 19
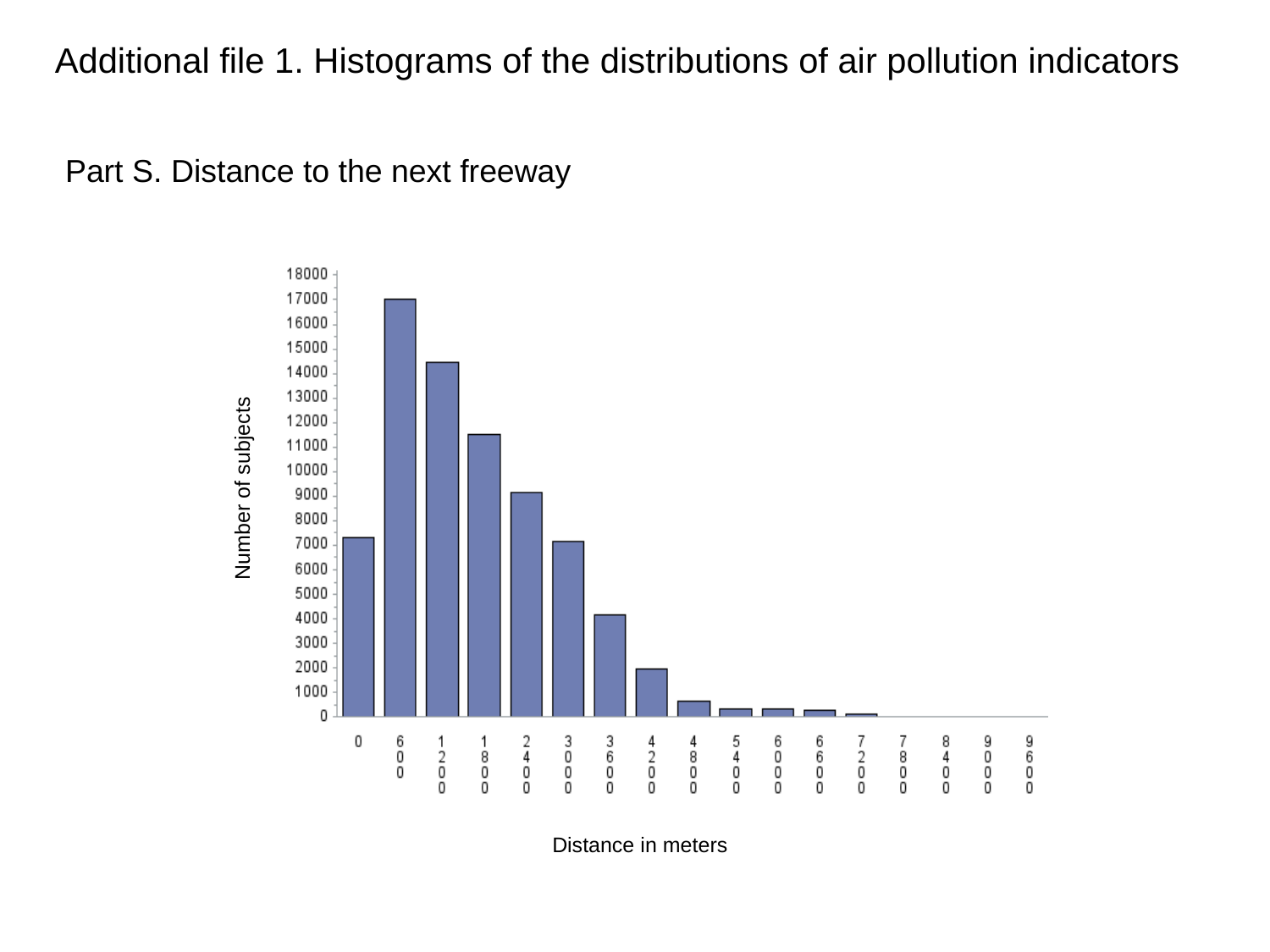

Additional file 1. Histograms of the distributions of air pollution indicators
Part S. Distance to the next freeway
Number of subjects
Distance in meters

## Slide 20
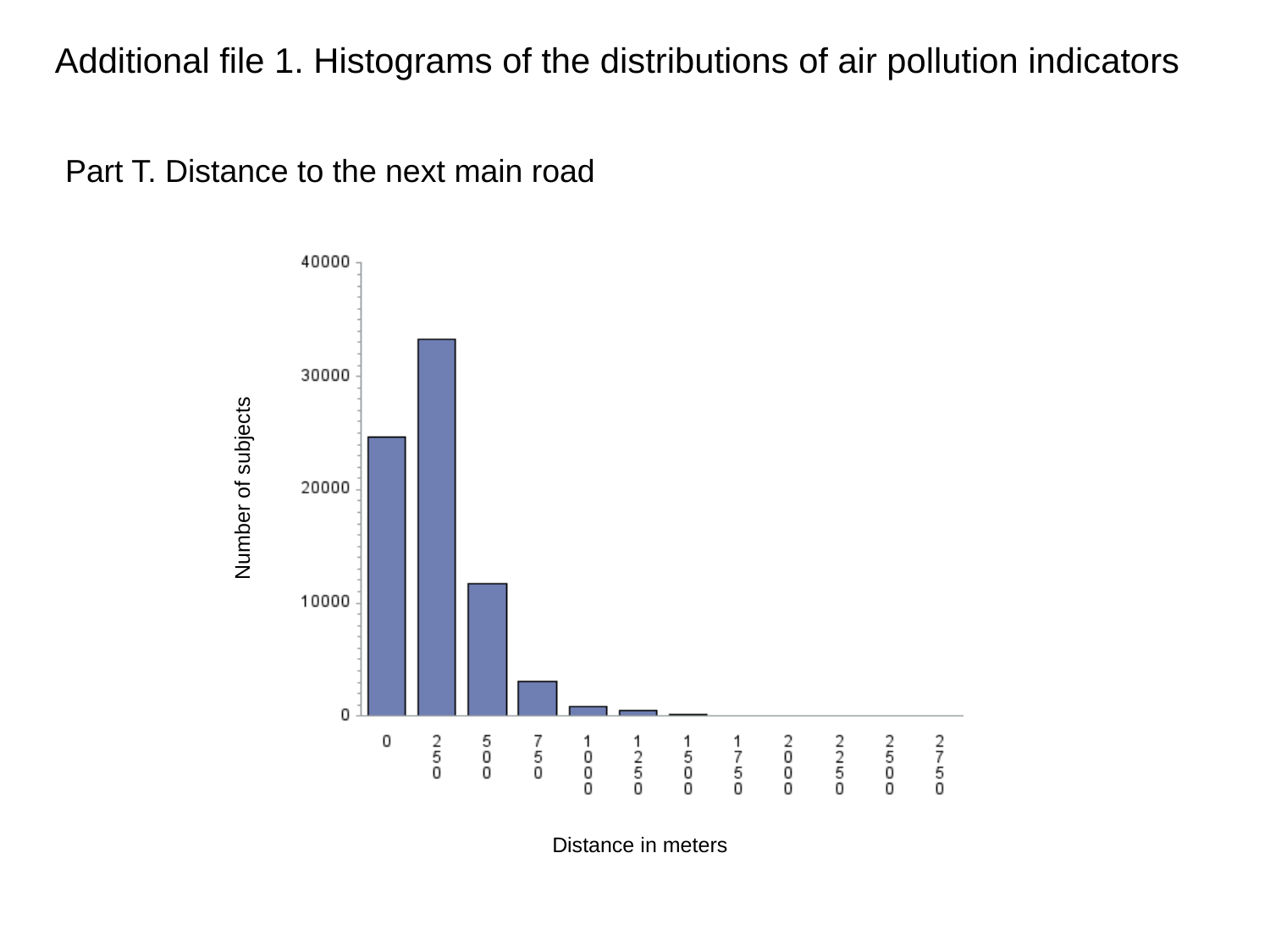

Additional file 1. Histograms of the distributions of air pollution indicators
Part T. Distance to the next main road
Number of subjects
Distance in meters
