# Supplementary material for: Investigating the association between birth weight and complementary air pollution metrics: a cohort study
Source: Environ Health. 2013 Feb 17;12:18. doi: 10.1186/1476-069X-12-18 (PMC3599912; doi:10.1186/1476-069X-12-18)
Supplement: Additional file 2 — associations between mean birth weight and an inter-quartile range in air pollution metrics - restricted populations, for pregnancy-long air pollution metrics. [file 1476-069X-12-18-S2.pdf]

Additional file 2. Sensitivity analyses : associations between mean birth weight and an inter-quartile range in air pollution metrics - restricted populations, for pregnancy-long air pollution metrics (a)

			Low birth weight				Mean birth weight (c)			
**Air pollution metrics**	**Interquartile range (IQR) in air pollution metrics (b)**	**Number of subjects**	**Odds ratio for IQR increase in air pollution metrics**	**95% confidence interval**		**p value**	**Change for IQR increase in air pollution metrics**	**95% confidence interval**		**p value**
**Monitoring station measurements, nearest station approach restricted to a 7 km radius (b)**										
**NO_2**	8.44	33254	0.92	0.82	1.03	0.15	24.53	17.91	31.16	< 0.01
**NO_x**	24.32	33380	0.95	0.87	1.05	0.30	19.38	13.67	25.10	< 0.01
**NO**	16.35	33254	0.97	0.89	1.05	0.43	15.74	10.58	20.90	< 0.01
**CO**	0.44	41000	0.97	0.89	1.06	0.54	18.20	12.84	23.56	< 0.01
**PM_{10}**	6.47	35055	0.98	0.88	1.09	0.68	13.32	7.48	19.16	< 0.01
**$PM_{2.5}$**	4.70	33694	0.93	0.82	1.05	0.24	18.84	11.89	25.80	< 0.01
**O_3**	10.87	41023	1.08	0.96	1.22	0.19	-27.96	-34.93	-20.98	< 0.01
**Traffic density, within buffers of different distances around roads, restricted to women residing within the buffers (b)**										
**50 m**	316.77	20030	1.04	0.91	1.19	0.56	2.79	-5.55	11.14	0.51
**75 m**	209.46	26860	1.05	0.95	1.16	0.34	-4.52	-10.73	1.68	0.15
**100 m**	149.98	35470	1.06	0.99	1.14	0.08	-3.03	-7.56	1.50	0.19
**150 m**	102.00	46969	1.07	1.02	1.12	< 0.01	-1.38	-4.41	1.65	0.37
**200 m**	88.41	54535	1.06	1.02	1.10	0.01	-0.65	-3.22	1.91	0.62
**250 m**	80.22	58568	1.04	1.00	1.08	0.06	-0.05	-2.38	2.28	0.97
**300 m**	73.29	61400	1.02	0.98	1.06	0.27	0.26	-1.88	2.41	0.81

a) adjusted for maternal age, length of gestation and poverty using smoothing splines and race/ethnicity, insurance, gender and parity as categorical variables : see results in table 3

b) the units are parts per million for CO, parts per billion for NO, NO_2 , NO_x , and O_3, and $\mu g.m^{-3}$ for PM_{10} and $PM_{2.5}$. Concentrations are averages, across the pregnancy period, derived from daily 24h- mean concentrations for NO_2, NO, NO_x, CO, PM_{10} and $PM_{2.5}$ and from daily mean concentrations from 10 am to 6 pm for O_3. The unit for traffic density is vehicle number per day/meter. The unit for distance to road is meters

c) in grams
