# Supplementary material for: Investigating the association between birth weight and complementary air pollution metrics: a cohort study
Source: Environ Health. 2013 Feb 17;12:18. doi: 10.1186/1476-069X-12-18 (PMC3599912; doi:10.1186/1476-069X-12-18)
Supplement: Additional file 3 — Sensitivity of the association between the air pollution metrics and birth outcomes to adjustment for hospital, county and time of conception. [file 1476-069X-12-18-S3.pdf]

Additional file 3. Sensitivity of the association between the air pollution metrics and birth outcomes to adjustment for hospital, county and time of conception

Low birth weight				Reference model (a), further adjusted for hospital				Reference model (a), further adjusted for county				Reference model (a), further adjusted for year and month of conception			
	Air pollution metrics	Interquartile range (IQR) in air pollution metrics (b)	Number of subjects	Odds ratio (c)	95% confidence interval		p value	Odds ratio (c)	95% confidence interval		p value	Odds ratio (c)	95% confidence interval		p value
Monitoring station measurements (d)	NO_2	11.87	68303	0.85	0.75	0.97	0.02	0.86	0.76	0.98	0.02	0.82	0.71	0.94	< 0.01
	NO_x	27.71	68303	0.95	0.87	1.04	0.31	0.96	0.87	1.05	0.32	0.92	0.83	1.02	0.11
	NO	17.90	68303	0.98	0.91	1.06	0.67	0.98	0.91	1.06	0.68	0.96	0.87	1.05	0.37
	CO	0.48	68303	0.98	0.90	1.07	0.71	0.98	0.90	1.07	0.72	0.97	0.88	1.07	0.6
	PM_{10}	6.76	68303	0.94	0.87	1.02	0.17	0.95	0.88	1.03	0.22	0.94	0.85	1.04	0.25
	$PM_{2.5}$	5.10	61623	0.94	0.85	1.05	0.26	0.95	0.86	1.05	0.36	0.86	0.74	0.99	0.04
	O_3	11.50	68303	1.09	0.96	1.23	0.17	1.09	0.96	1.24	0.17	1.16	1.02	1.31	0.02
LUR	NO_2	9.34	68303	0.94	0.86	1.02	0.16	0.94	0.87	1.02	0.15	0.95	0.85	1.07	0.39
	NO_x	25.24	68303	0.98	0.91	1.06	0.65	0.98	0.91	1.05	0.57	1.01	0.91	1.12	0.78
CALINE4	NO_x	5.65	67043	0.97	0.90	1.05	0.48	0.98	0.91	1.05	0.56	0.98	0.91	1.06	0.64
	CO	0.08	67043	0.97	0.90	1.05	0.45	0.98	0.91	1.05	0.53	0.98	0.91	1.06	0.64
	$PM_{2.5}$	1.36	67043	0.99	0.92	1.06	0.70	0.99	0.93	1.06	0.82	0.98	0.92	1.05	0.66
Traffic density (e)	50 m	12.93	68303	1.00	1.00	1.01	0.56	1.00	1.00	1.01	0.53	1.00	1.00	1.01	0.56
	75 m	35.91	68303	1.01	0.99	1.02	0.26	1.01	0.99	1.02	0.24	1.01	0.99	1.02	0.26
	100 m	53.91	68303	1.02	1.00	1.05	0.06	1.02	1.00	1.05	0.06	1.02	1.00	1.05	0.06
	150 m	74.30	68303	1.05	1.01	1.08	0.01	1.05	1.01	1.08	< 0.01	1.04	1.01	1.08	0.01
	200 m	84.35	68303	1.05	1.01	1.09	0.01	1.05	1.01	1.09	0.01	1.05	1.01	1.09	0.01
	250 m	81.34	68303	1.04	1.00	1.08	0.05	1.04	1.00	1.08	0.05	1.04	1.00	1.08	0.06
	300 m	76.58	68303	1.02	0.99	1.06	0.20	1.03	0.99	1.06	0.19	1.02	0.99	1.06	0.21
Distance to the nearest road	Freeways	1766.81	68303	1.04	0.95	1.14	0.36	1.04	0.95	1.13	0.38	1.04	0.96	1.14	0.35
	Major roads	253.05	68303	0.93	0.87	1.00	0.05	0.93	0.87	1.00	0.05	0.94	0.87	1.01	0.07

a) adjusted for maternal age, length of gestation and poverty using smoothing splines and race/ethnicity, insurance, gender and parity as categorical variables : see results in table 3

b) the units are parts per million for CO, parts per billion for NO, NO_2 , NO_x , and O_3, and µg.m^{-3} for PM_{10} and $PM_{2.5}$. Concentrations are averages, across the pregnancy period, derived from daily 24h- mean concentrations for NO_2, NO, NO_x, CO, PM_{10} and $PM_{2.5}$ and from daily mean concentrations from 10 am to 6 pm for O_3. The unit for traffic density is vehicle number per day/meter. The unit for distance to road is meters

c) for an interquartile range increase in air pollution metrics

d) nearest station approach without distance restrictions

e) within buffers of different distances around roads

Additional file 3 (continued). Sensitivity of the association between the air pollution metrics and birth outcomes to adjustment for hospital, county and time of conception

**Mean birth weight**				**Reference model (a), further adjusted for hospital**				**Reference model (a), further adjusted for county**				**Reference model (a), further adjusted for year and month of conception**			
	**Air pollution metrics**	**Interquartile range (IQR) in air pollution metrics (b)**	**Number of subjects**	**Change (c)**	**95% confidence interval**		**p value**	**Change (c)**	**95% confidence interval**		**p value**	**Change (c)**	**95% confidence interval**		**p value**
**Monitoring station measurements (d)**	**NO_2**	11.87	68303	33.18	25.79	40.57	< 0.01	31.10	24.08	38.12	< 0.01	30.85	23.61	38.09	< 0.01
	**NO_x**	27.70	68303	19.42	14.15	24.69	< 0.01	19.83	14.59	25.07	< 0.01	22.49	16.77	28.20	< 0.01
	**NO**	17.90	68303	14.20	9.65	18.75	< 0.01	14.73	10.16	19.29	< 0.01	17.86	12.74	22.98	< 0.01
	**CO**	0.48	68303	17.12	11.96	22.29	< 0.01	17.11	12.15	22.07	< 0.01	16.76	11.29	22.23	< 0.01
	**PM_{10}**	6.76	68303	17.92	13.31	22.53	< 0.01	17.09	12.88	21.29	< 0.01	14.94	9.47	20.40	< 0.01
	**$PM_{2.5}$**	5.10	61623	21.96	15.94	27.97	< 0.01	21.87	16.30	27.45	< 0.01	27.36	19.59	35.13	< 0.01
	**O_3**	11.50	68303	-23.48	-30.38	-16.57	< 0.01	-25.55	-32.72	-18.38	< 0.01	-28.84	-35.61	-22.08	< 0.01
**LUR**	**NO_2**	9.34	68303	17.26	12.56	21.96	< 0.01	16.11	11.52	20.70	< 0.01	4.58	-1.51	10.66	0.14
	**NO_x**	25.24	68303	10.70	6.53	14.89	0.03	10.89	6.71	15.07	0.01	-2.39	-7.95	3.17	0.40
**CALINE4**	**NO_x**	5.65	67043	11.42	7.34	15.49	< 0.01	10.82	6.88	14.76	< 0.01	9.25	5.27	13.23	< 0.01
	**CO**	0.08	67043	12.18	8.07	16.28	< 0.01	11.60	7.63	15.56	< 0.01	9.59	5.54	13.64	< 0.01
	**$PM_{2.5}$**	1.36	67043	4.67	0.83	8.51	0.02	4.31	0.67	7.95	0.02	7.33	3.68	10.98	< 0.01
**Traffic density (e)**	**50 m**	12.91	68303	0.06	-0.21	0.32	0.68	0.06	-0.21	0.33	0.66	0.09	-0.18	0.35	0.53
	**75 m**	35.91	68303	-0.21	-1.12	0.70	0.64	-0.20	-1.10	0.71	0.67	-0.10	-1.01	0.80	0.82
	**100 m**	53.91	68303	-0.56	-2.05	0.92	0.46	-0.52	-2.00	0.96	0.49	-0.36	-1.84	1.12	0.64
	**150 m**	74.30	68303	-0.60	-2.69	1.49	0.57	-0.52	-2.60	1.57	0.63	-0.18	-2.27	1.90	0.86
	**200 m**	84.33	68303	-0.36	-2.73	2.01	0.77	-0.26	-2.61	2.10	0.83	0.19	-2.17	2.55	0.87
	**250 m**	81.34	68303	-0.10	-2.41	2.20	0.93	-0.01	-2.30	2.28	0.99	0.43	-1.86	2.73	0.71
	**300 m**	76.57	68303	0.24	-1.97	2.45	0.83	0.33	-1.86	2.53	0.77	0.73	-1.46	2.93	0.51
**Distance to the nearest road**	**Freeways**	1766.80	68303	-8.68	-13.40	-3.96	< 0.01	-7.24	-11.94	-2.54	< 0.01	-7.19	-11.89	-2.49	< 0.01
	**Major roads**	253.05	68303	-0.34	-3.83	3.15	0.85	-0.38	-3.86	3.10	0.83	-1.78	-5.24	1.69	0.32

a) adjusted for maternal age, length of gestation and poverty using smoothing splines and race/ethnicity, insurance, gender and parity as categorical variables : see results in table 3
b) the units are parts per million for CO, parts per billion for NO, NO_2 , NO_x , and O_3, and $\mu g.m^{-3}$ for PM_{10} and $PM_{2.5}$. Concentrations are averages, across the pregnancy period, derived from daily 24h- mean concentrations for NO_2, NO, NO_x, CO, PM_{10} and $PM_{2.5}$ and from daily mean concentrations from 10 am to 6 pm for O_3. The unit for traffic density is vehicle number per day/meter. The unit for distance to road is meters
c) for an interquartile range increase in air pollution metrics
d) nearest station approach without distance restrictions
e) within buffers of different distances around roads
