# Supplementary material for: Investigating the association between birth weight and complementary air pollution metrics: a cohort study
Source: Environ Health. 2013 Feb 17;12:18. doi: 10.1186/1476-069X-12-18 (PMC3599912; doi:10.1186/1476-069X-12-18)
Supplement: Additional file 4 — associations between birth weight and an inter-quartile range in air pollution metrics, for pregnancy-long exposures, without adjustment for the length of gestation. [file 1476-069X-12-18-S4.pdf]

Additional file 4. Sensitivity analyses: associations between birth weight and an inter-quartile range in air pollution metrics, for pregnancy-long exposures (a), without adjustment for the length of gestation

Air pollution metrics	IQR in air pollution metrics (c)	Number of subjects	Low birth weight<br>Odds ratio for an IQR increase in air pollution metrics (c)	95% confidence interval		p value	Mean birth weight (d)<br>Change for an IQR increase in air pollution metrics (c)	95% confidence interval		p value
**Monitoring station measurements, nearest station approach without distance restrictions (b)**										
**NO_2**	11.87	68,303	0.86	0.77	0.95	0.86	34.83	28.75	40.91	< 0.01
**NO_x**	27.70	68,303	0.93	0.86	1.01	0.09	24.24	19.50	28.97	< 0.01
**NO**	17.90	68,303	0.96	0.90	1.03	0.28	19.39	15.11	23.68	< 0.01
**CO**	0.48	68,303	0.96	0.89	1.04	0.36	21.93	17.10	26.76	< 0.01
**PM_{10}**	6.76	68,303	0.94	0.88	1.02	0.13	19.14	14.84	23.44	< 0.01
**$PM_{2.5}$**	5.10	61,623	0.94	0.85	1.03	0.17	25.06	19.47	30.65	< 0.01
**O_3**	11.50	68,303	1.12	1.01	1.23	0.03	-30.20	-35.99	-<br>24.41	< 0.01
Land Use Regression model predictions (b)										
**NO_2**	9.34	68,303	0.94	0.86	1.01	0.11	16.90	12.05	21.75	< 0.01
**NO_x**	25.24	68,303	0.98	0.90	1.05	0.54	10.49	6.08	14.90	< 0.01
**CALINE4 predictions (b)**										
**NO_x**	5.65	67,043	0.97	0.91	1.04	0.37	13.93	9.88	17.98	< 0.01
**CO**	0.08	67,043	0.96	0.89	1.03	0.27	14.85	10.79	18.91	< 0.01
**$PM_{2.5}$**	1.36	67,043	0.98	0.92	1.05	0.57	5.42	1.58	9.26	0.01
Traffic density, within buffers of different distances around roads (b)										
**50 m**	12.91	68,303	1.00	1.00	1.01	0.49	0.01	-0.28	0.29	0.96
**75 m**	35.91	68,303	1.01	0.99	1.02	0.23	-0.38	-1.34	0.58	0.44
**100 m**	53.91	68,303	1.02	1.00	1.04	0.07	-0.68	-2.25	0.89	0.40
**150 m**	74.30	68,303	1.04	1.01	1.07	0.01	-0.36	-2.57	1.85	0.75
**200 m**	84.33	68,303	1.04	1.00	1.08	0.03	0.42	-2.08	2.91	0.74
**250 m**	81.34	68,303	1.03	0.99	1.07	0.16	0.93	-1.50	3.36	0.45
**300 m**	76.57	68,303	1.01	0.98	1.05	0.46	1.32	-1.00	3.65	0.26
**Air pollution metrics**	**IQR in air pollution**	**Number of**	**Low birth weight**<br>**Odds ratio for an IQR**	**95% confide**		**p value**	**Mean birth weight (d)**<br>**Change for an IQR increase in air**	**95% confidence interval**		**p value**

	metrics (c)	subjects	increase in air pollution metrics (c)	nce interval			pollution metrics (c)			
Distance to the nearest road (b)										
**Freeways**	1766.80	68,303	1.05	0.96	1.14	0.28	-7.54	-12.52	-2.56	< 0.01
**Major roads**	253.05	68,303	0.93	0.87	1.00	0.06	-1.57	-5.24	2.10	0.27

a) adjusted for maternal age and poverty using smoothing splines and maternal race/ethnicity, insurance, parity, and gender of the infant as categorical variables

b) the units are parts per million for CO, parts per billion for NO, NO_2 , NO_x , and O_3, and µg.m^{-3} for PM_{10} and $PM_{2.5}$. Concentrations are averages, across the pregnancy period, derived from daily 24h- mean concentrations for NO_2, NO, NO_x, CO, PM_{10} and $PM_{2.5}$ and from daily mean concentrations from 10 am to 6 pm for O_3. The unit for traffic density is vehicle number per day/meter. The unit for distance to road is meters

c) IQR stands for inter-quartile range

d) in grams
