# Supplementary material for: Investigating the association between birth weight and complementary air pollution metrics: a cohort study
Source: Environ Health. 2013 Feb 17;12:18. doi: 10.1186/1476-069X-12-18 (PMC3599912; doi:10.1186/1476-069X-12-18)
Supplement: Additional file 5 — Associations between mean birth weight and an inter-quartile range in monitoring stations measurements, by pregnancy trimester. [file 1476-069X-12-18-S5.pdf]

Additional file 5. Associations between mean birth weight and an inter-quartile range in monitoring stations measurements, by pregnancy trimester (a)

Air pollution e metrics	Interquartile range (IQR) in air pollution metrics(b)	Number of subjects	Low birth weight: Odds ratio for IQR increase in air pollution metrics	Low birth weight: 95% confidence interval		Low birth weight: p value	Mean birth weight (c): Change for IQR increase in air pollution metrics	Mean birth weight (c): 95% confidence interval		Mean birth weight (c): p value
**NO_2, first trimester**	13.80	68303	0.92	0.84	1.00	0.06	17.18	12.53	21.83	< 0.01
**NO_2, second trimester**	14.10	68303	0.88	0.80	0.96	< 0.01	24.62	19.84	29.41	< 0.01
**NO_2, third trimester**	14.12	68303	0.93	0.86	1.02	0.13	20.92	16.11	25.73	< 0.01
**NO_x, first trimester**	51.36	68303	0.96	0.89	1.04	0.33	10.08	5.52	14.63	< 0.01
**NO_x, second trimester**	49.52	68303	0.93	0.86	1.01	0.1	18.79	14.22	23.36	< 0.01
**NO_x, third trimester**	48.72	68303	0.98	0.91	1.06	0.62	14.41	9.91	18.91	< 0.01
**NO, first trimester**	40.12	68303	0.97	0.90	1.06	0.53	7.54	2.91	12.16	< 0.01
**NO, second trimester**	37.63	68303	0.95	0.88	1.03	0.24	16.37	11.81	20.94	< 0.01
**NO, third trimester**	36.84	68303	0.99	0.92	1.07	0.88	11.96	7.49	16.43	< 0.01
**CO, first trimester**	0.64	68303	0.98	0.91	1.05	0.52	11.06	6.82	15.30	< 0.01
**CO, second trimester**	0.62	68303	0.97	0.90	1.04	0.39	18.34	14.09	22.58	< 0.01
**CO, third trimester**	0.59	68303	0.97	0.91	1.04	0.45	14.47	10.43	18.51	< 0.01
**PM_{10}, first trimester**	9.29	68303	0.96	0.89	1.04	0.32	15.45	11.32	19.58	< 0.01
**PM_{10}, second trimester**	9.18	68303	0.95	0.88	1.02	0.17	19.19	14.97	23.42	< 0.01
**PM_{10}, third trimester**	9.11	68303	0.95	0.88	1.02	0.16	14.45	10.19	18.71	< 0.01
**$PM_{2.5}$, first trimester**	6.71	60907	0.98	0.90	1.07	0.71	15.32	10.62	20.02	< 0.01
**$PM_{2.5}$, second trimester**	6.47	62247	0.93	0.86	1.02	0.11	18.99	14.35	23.63	< 0.01
**$PM_{2.5}$, third trimester**	6.28	63624	0.94	0.86	1.02	0.11	15.36	10.84	19.89	< 0.01
**O_3, first trimester**	17.78	68303	1.03	0.94	1.13	0.54	-13.52	-18.69	-8.34	< 0.01
**O_3, second trimester**	17.46	68303	1.12	1.02	1.23	0.02	-23.53	-28.73	-18.32	< 0.01
**O_3, third trimester**	17.43	68303	1.07	0.98	1.17	0.15	-17.82	-22.98	-12.66	< 0.01

a) adjusted for maternal age, length of gestation and poverty using smoothing splines and race/ethnicity, insurance, gender and parity as categorical variables : see results in table 3

b) the units are parts per million for CO, parts per billion for NO, NO_2, NO_x , and O_3, and µg.m^{-3} for PM_{10} and $PM_{2.5}$. Concentrations are averages, across each pregnancy trimester, derived from daily 24h- mean concentrations for NO_2 , NO, NO_x, CO, PM_{10} and $PM_{2.5}$ and from daily mean concentrations from 10 am to 6 pm for O_3. The unit for traffic density is vehicle number per day/meter. The unit for distance to road is meters

c) in grams
