# Supplementary material for: Investigating the association between birth weight and complementary air pollution metrics: a cohort study
Source: Environ Health. 2013 Feb 17;12:18. doi: 10.1186/1476-069X-12-18 (PMC3599912; doi:10.1186/1476-069X-12-18)
Supplement: Additional file 6 — Part A. Mean change in birth weight according to the distance to the nearest freeway (restricted to distances between 0 and 1000 m). [file 1476-069X-12-18-S6.ppt]

## Slide 1
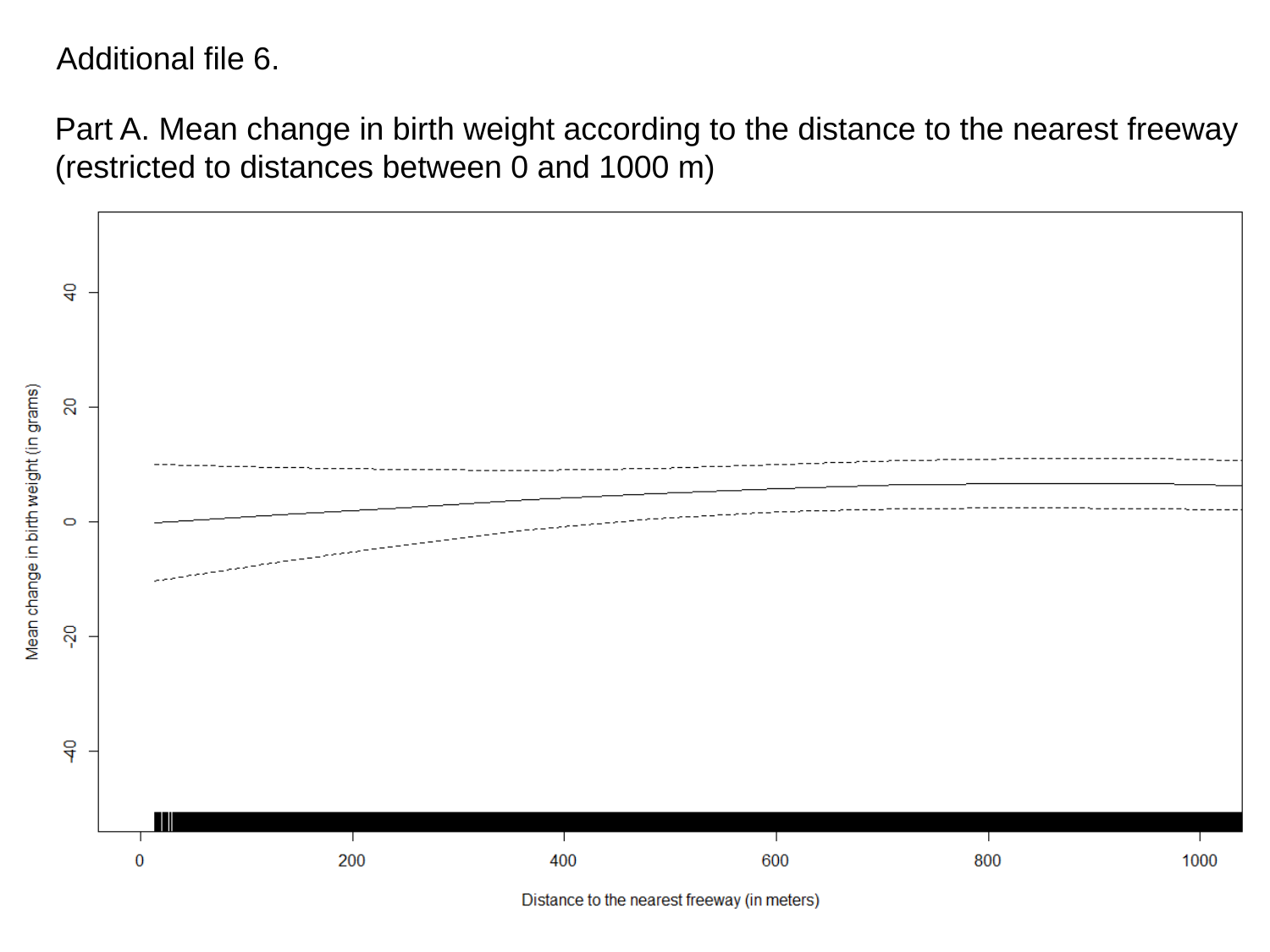

Additional file 6.
Part A. Mean change in birth weight according to the distance to the nearest freeway (restricted to distances between 0 and 1000 m)
#

## Slide 2
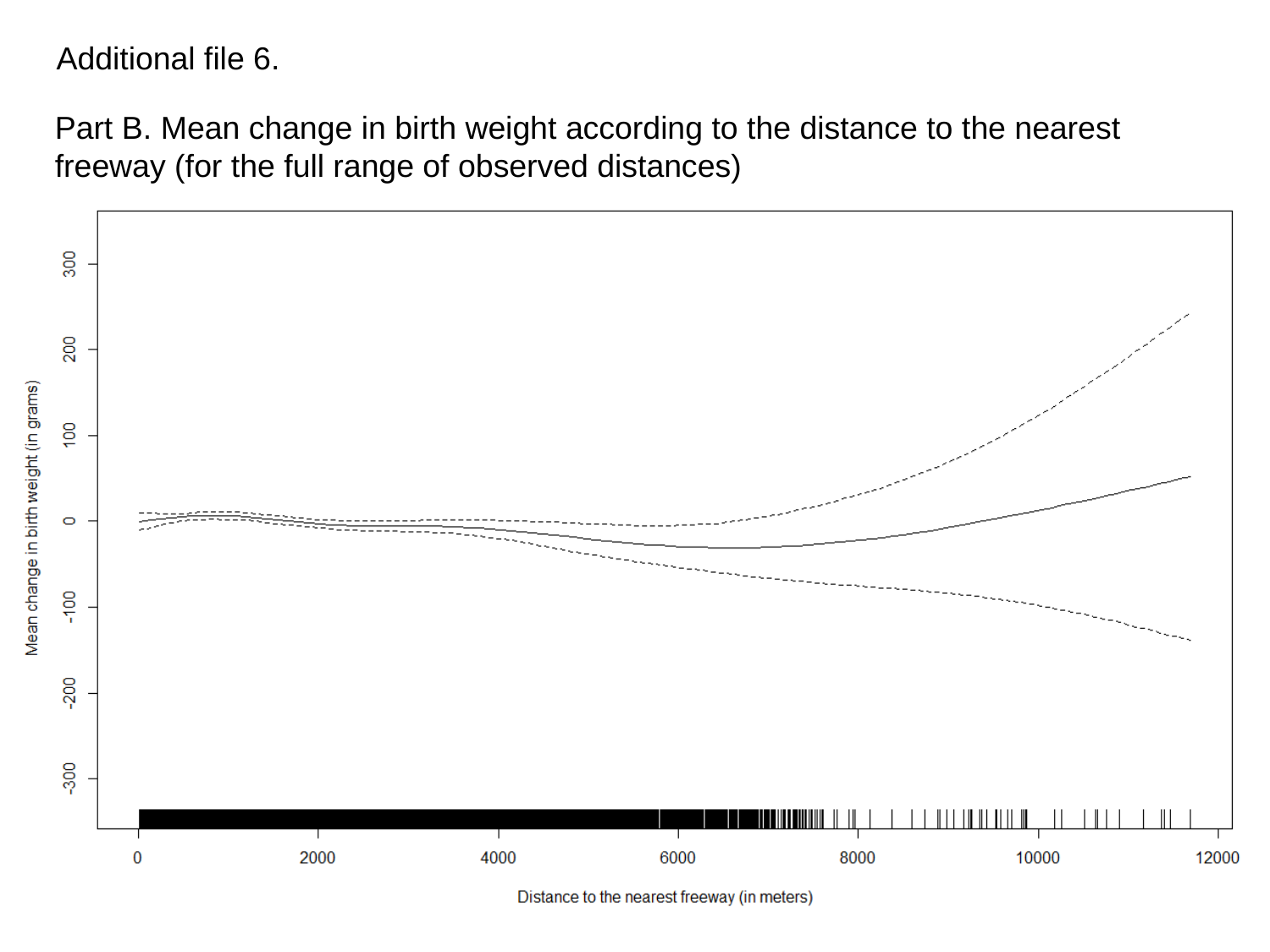

Additional file 6.
Part B. Mean change in birth weight according to the distance to the nearest freeway (for the full range of observed distances)
