# Supplementary material for: Investigating the association between birth weight and complementary air pollution metrics: a cohort study
Source: Environ Health. 2013 Feb 17;12:18. doi: 10.1186/1476-069X-12-18 (PMC3599912; doi:10.1186/1476-069X-12-18)
Supplement: Additional file 7 — Descriptive statistics for term births in Los Angeles and Orange Counties birth certificates (2001) and in the Memorial Care database (1997-2006). [file 1476-069X-12-18-S7.pdf]

Additional file 7. Descriptive statistics for term births in Los Angeles and Orange Counties birth certificates (2001) and in the Memorial Care database (1997-2006).

Table 7A. Descriptive statistics for maternal race/ethnicity in Los Angeles and Orange Counties birth certificates (2001) and in the Memorial Care database (1997-2006) - term births only.

**Los Angeles and Orange counties**		
	**Birth certificates (2001)**	**Memorial database (1997-2006)**
**Maternal race/ethnicity**	Percent	Percent
Caucasian	22.25	41.05
Hispanic	59.60	31.82
African American	5.78	8.41
Asian	10.75	9.89
Other	1.35	4.59
Missing	0.28	4.24
**Los Angeles county**		
	**Birth certificates (2001)**	**Memorial database (1997-2006)**
**Maternal race/ethnicity**	Percent	Percent
Caucasian	18.64	26.66
Hispanic	62.69	38.01
African American	7.18	16.45
Asian	9.89	9.91
Other	1.38	5.38
Missing	0.23	3.59
**Orange county**		
	**Birth certificates (2001)**	**Memorial database (1997-2006)**
**Maternal race/ethnicity**	Percent	Percent
Caucasian	34.70	53.67
Hispanic	48.95	26.39
African American	0.97	1.37
Asian	13.70	9.87
Other	1.24	3.90
Missing	0.45	4.81

Table 7B. Descriptive statistics for maternal insurance status in Los Angeles and Orange Counties birth certificates (2001) and in the Memorial Care database (1997-2006) - term births only.

**Los Angeles and Orange counties**		
	**Birth certificates (2001)**	**Memorial database (1997-2006)**
**Maternal insurance status**	Percent	Percent
Public	0.46	0.28
Private	0.51	0.68
Missing	0.02	0.04

**Los Angeles county**		
	**Birth certificates (2001)**	**Memorial database (1997-2006)**
**Maternal insurance status**	Percent	Percent
Public	48.99	42.21
Private	48.65	57.25
Missing	2.36	0.54

**Orange county**		
	**Birth certificates (2001)**	**Memorial database (1997-2006)**
**Maternal insurance status**	Percent	Percent
Public	36.60	14.75
Private	60.80	78.13
Missing	2.60	7.12

Table 7C. Proportions of low birth weight infants in Los Angeles and Orange Counties birth certificates (2001) and in the Memorial Care database (1997-2006) - term births only.

**Los Angeles and Orange counties**		
	**Birth certificates (2001)**	**Memorial database (1997-2006)**
**Birth weight <2,500 g**	Percent	Percent
Yes	2.55	1.71
No	97.45	98.29

**Los Angeles county**		
	**Birth certificates (2001)**	**Memorial database (1997-2006)**
**Birth weight <2,500 g**	Percent	Percent
Yes	2.64	1.5
No	97.36	98.5

**Orange county**		
	**Birth certificates (2001)**	**Memorial database (1997-2006)**
**Birth weight <2,500 g**	Percent	Percent
Yes	2.23	1.94
No	97.77	98.06
